# Enhancing data pipelines for forecasting student performance: integrating feature selection with cross-validation

**DOI:** 10.1186/s41239-021-00279-6

**Published:** 2021-08-17

**Authors:** Roberto Bertolini, Stephen J. Finch, Ross H. Nehm

**Affiliations:** 1 Department of Applied Mathematics and Statistics, Stony Brook University, Math Tower, Room P-139A, Stony Brook, NY 11794-3600 USA; 2 Department of Ecology and Evolution, Program in Science Education, Stony Brook University, 650 Life Sciences Building, Stony Brook, NY 11794-5233 USA

**Keywords:** Data pipeline, Feature selection, Cross-validation, Data mining, Introductory biology

## Abstract

Educators seek to harness knowledge from educational corpora to improve student performance outcomes. Although prior studies have compared the efficacy of data mining methods (DMMs) in pipelines for forecasting student success, less work has focused on identifying a set of relevant features prior to model development and quantifying the stability of feature selection techniques. Pinpointing a subset of pertinent features can (1) reduce the number of variables that need to be managed by stakeholders, (2) make “black-box” algorithms more interpretable, and (3) provide greater guidance for faculty to implement targeted interventions. To that end, we introduce a methodology integrating feature selection with cross-validation and rank each feature on subsets of the training corpus. This modified pipeline was applied to forecast the performance of 3225 students in a baccalaureate science course using a set of 57 features, four DMMs, and four filter feature selection techniques. Correlation Attribute Evaluation (CAE) and Fisher’s Scoring Algorithm (FSA) achieved significantly higher Area Under the Curve (AUC) values for logistic regression (LR) and elastic net regression (GLMNET), compared to when this pipeline step was omitted. Relief Attribute Evaluation (RAE) was highly unstable and produced models with the poorest prediction performance. Borda’s method identified grade point average, number of credits taken, and performance on concept inventory assessments as the primary factors impacting predictions of student performance. We discuss the benefits of this approach when developing data pipelines for predictive modeling in undergraduate settings that are more interpretable and actionable for faculty and stakeholders.

**Supplementary Information:**

The online version contains supplementary material available at 10.1186/s41239-021-00279-6.

## Introduction

**Fig. 1:**
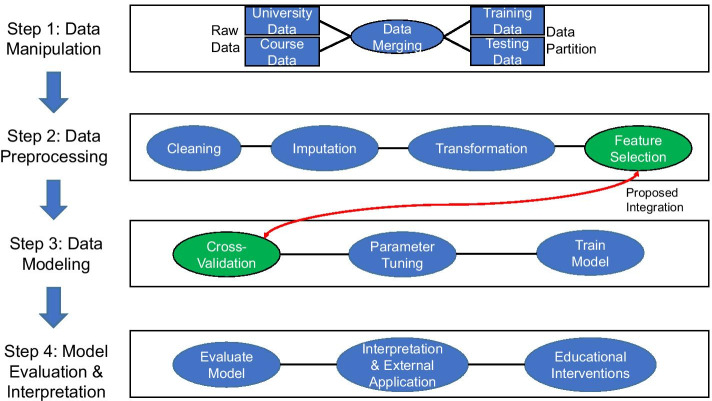
Overview of a standard data science pipeline and proposed integration of feature selection with cross-validation

Educational data mining (EDM) focuses on developing mathematical frameworks for analyzing large educational corpora (Baker, 2010, 2014). This field has grown to focus on predicting the success of students in various instructional settings from individual courses to entire educational systems (Costa et al., 2017; Knowles, 2015; Schwarzenberg et al., 2020; Silva & Fonseca, 2017). A concentration of research is focused on addressing educational concerns and detecting at-risk students in the learning environment through the development of data pipelines to make forecasts of student retention and attrition (Beck & Davidson, 2001; Burgos et al., 2018; Chang et al., 2014; ECAR-Analytics Working Group, 2015; Gašević et al., 2016; Griff & Matter, 2008; Herzog, 2006; Olivé et al., 2020; Yu et al., 2010). Data science pipelines (Fig. 1) are a sequence of computing steps undertaken to assemble, process, and model data corpora records (Rupprecht et al., 2020; Shang et al., 2019; Skiena, 2017). Specific examples of the use of data pipelines in education include the development of early warning systems (EWS), computational systems to track, monitor, and predict student performance (Howard et al., 2018; Hu et al., 2014), and the design of system architecture to expedite data assembly and modeling for stakeholders (Ansari et al., 2017; Guruler et al., 2010). Data mining methods (DMMs) have enhanced the accuracy of these pipelines due to their ability to extract complex patterns and generate knowledge from large corpora (Rovira et al., 2017).

A critical component of EDM is assembling a collection of features (i.e., covariates, independent variables) that can be used to make evidence-based administrative and pedagogical decisions in order to improve the quality of student success and university life (Tekin, 2014). In a large corpus, only a selection of available features tends to be associated with the dependent variable of interest (e.g., course grade, an indicator of on-track graduation status). The remaining features may not be meaningfully informative and increase the magnitude of data that needs to be managed by pipelines, stored by the institution, and analyzed or interpreted.

Feature selection is the process of selecting a subset of all available features in a corpus to enhance the efficacy of prediction models (Chandrashekar & Sahin, 2014; Koller & Sahami, 1996). As the number of features increases, the problem of finding an optimal subset that models the target outcome becomes intractable because it is computationally impractical to evaluate all possible subsets of features (Kohavi and John, 1997). Therefore, sub-optimal screening methodologies (i.e., feature selection techniques) have been developed to flag and omit extraneous features from the final prediction model. If feature selection is poorly performed and erroneously omits features associated with the target outcome, then poor performance on independent corpora and inaccurate predictions in a large data science pipeline may occur (Urbanowicz et al., 2018). Moreover, identifying a set of informative variables can reduce data storage, reduce the complexity of prediction models, and aid in interpretability for stakeholders (Brooks & Thompson, 2017; López-Zambrano et al., 2020).

Feature selection can be applied when evaluating a prediction model (Chong & Jun, 2005; Xie et al., 2020) or during data preprocessing (Hancer et al., 2018; Talavera, 1999; Yu & Liu, 2003). The latter approach is more beneficial since reducing the number of features prior to constructing prediction models in pipelines may (1) reduce the complexity of pipelines, (2) allow educational stakeholders to assemble, store, and work with a smaller set of features, and (3) make esoteric ‘black-box’ prediction algorithms more interpretable.

When training prediction models in data science pipelines, DMMs are generally run on different subsets of the training corpus to tune hyperparameters and build prediction models. To reduce overfitting and enable DMMs to generalize to independent corpora, cross-validation is traditionally used. This standard technique in the computational sciences quantifies the differential efficacy of several candidate prediction algorithms for model validation and selection (Alpaydin, 2020; Arlot & Celisse, 2010; Kohavi, 1995; Shao, 1993). During this procedure, the training data are partitioned into *k* subsamples called folds (Allen, 1974; Arlot & Lerasle, 2016; Geisser, 1975). Each fold is used once as a validation set while the remaining *k*-1 folds encompass the training set. Other alternatives for model selection (e.g., hold-out, resampling methodologies) limit the number of observations used to train the DMMs and are computationally intensive (Hawkins et al., 2003; Skiena, 2017; Xiong et al., 2020). Since data features impact the performance of DMMs during training, it is imperative to ascertain the importance of different features to quantify variability in predictive efficacy at this critical juncture of the pipeline and identify candidate features impacting the performance of prediction algorithms.

Therefore, integrating feature selection with cross-validation may potentially simplify the data pipeline and make DMMs more interpretable. However, in order to quantify the association between features and the target outcome on subsets of the training data, a systematic consensus ranking system is needed to tabulate the merit of each feature across different data subsets. Moreover, a metric of stability is needed to quantify the robustness of the set of pertinent features identified on different subsets of the corpus to discern how similar these features are and how they affect predictive efficacy.

In this study, we introduce a methodology integrating feature selection with the cross-validation step of data pipelines, as well as devise a consensus ranking scheme to compare the sets of impactful features on subsets of the data using a selection of filter feature selection techniques. Unlike other feature selection techniques (i.e., wrapper and embedded methods), filter methods are independent of the DMM (Bolón-Canedo et al., 2013). The three overarching goals of this study are to (1) present a modified and simplified data pipeline, (2) examine whether the use of filter feature selection techniques improve forecast accuracy when generating predictions of student success in undergraduate biology, and (3) identify sets of academic and non-academic features that contribute to student performance prior to model training. After introducing our research questions, we discuss how our methodology can address limitations in prior feature selection EDM literature, before presenting our integrated pipeline and research context. We conclude with a discussion on how this methodology can make computational tools more interpretable for faculty and stakeholders and guide them in the development of psychosocial support structures and educational interventions to foster student success.

## Research questions

This study addressed the following three research questions:

(RQ 1) Do preprocessing feature selection techniques enhance the predictive efficacy of DMMs compared to when this step is omitted from the EDM pipeline?

(RQ 2) How consistent are the relevant features identified by the preprocessing feature selection techniques on subsets of the training data?

(RQ 3) What features do different feature selection techniques identify as contributing factors to student performance in the collegiate biology classroom, providing interpretable and actionable information for faculty and stakeholders?

## Literature review

EDM studies generally identify the most important features in pipelines after the prediction model has been fully developed using the Gini index to rank highly pertinent features (for examples see Hardman et al., 2013; Alexandro, 2018; Xue, 2018; Berens et al., 2019). Aside from the fact that this index tends to favor continuous features and categorical features with many levels, all features are used in their final models to predict student performance outcomes. This can potentially reduce overall classification accuracy due to model overfitting (Breiman et al., 1984; Strobl et al., 2007). We review EDM research applying feature selection as a preprocessing step in pipelines below and address how integrating feature selection with cross-validation to rank features by their association with the target outcome can address the limitations in these studies.

Several EDM studies only use one preprocessing feature selection technique, such as chi-square attribute evaluation to rank features by their chi-squared statistic (e.g., Bucos & Drăgulescu, 2018; Kovačić, 2010). Kovačić (2010) used three decision tree algorithms to predict student attrition using demographics and collegiate course performance records at a New Zealand college. The authors identified (1) ethnicity, (2) course program, and (3) course block as the most impactful features. Bucos and Drăgulescu (2018) focused on using five DMMs to model course performance in Romania. All DMMs yielded similar area under the curve (AUC) values, ranging between 0.81 and 0.83.

More detailed work has examined several preprocessing feature selection techniques. Ramaswami and Bhaskaran (2009) applied six feature selection techniques to model secondary school performance using student demographics and 4 DMMs. Correlation attribute evaluation (CAE) and information gain attribute evaluation (IG) achieved the highest AUC (0.729), selecting nine and seven features out of 32 possible ones, respectively. Márquez-Vera et al. (2016) used 10 preprocessing feature selection techniques and six DMMs to predict high school class performance in Mexico at seven distinct time points during a course. Their research was divided into three sections: (1) DMMs and feature selection techniques were run on all aggregated features starting from pre-course, (2) models using a limited set of features appearing at least twice during ten-fold cross-validation, across all feature selection techniques, were used to train separate models at each time frame, and (3) training corpora were balanced using an oversampling technique entitled SMOTE (Synthetic Minority Over-sampling Technique) (Chawla et al., 2002). The third scenario achieved the highest predictive accuracy, across all time points, compared to when preprocessing feature selection techniques were omitted from the data pipeline.

There are several limitations to these prior EDM studies. First, they do not provide a mathematical framework to compare the performance of the preprocessing feature selection techniques across independent corpora and assess whether the features identified are similar across different methods. While Márquez-Vera et al. (2016) selected the top-tiered features in their corpora during cross-validation, their method requires the researcher to apply 10 feature selection techniques to the data simultaneously. Ranking features using a single method would simplify the data science pipeline considerably. Furthermore, when ranking features, these EDM studies used a threshold or specified a predetermined number of features to include in the final model. This is also a common practice in other disciplines outside of EDM (Osman et al., 2017; Rachburee & Punlumjeak, 2015). However, in applied studies, it is unknown how many features should be used for developing prediction models. Research has offered little guidance on the number of features to select, except for Khoshgoftaar et al. (2007) who found that ${\mathrm{log}}_{2}(m)$—features (rounded up to the nearest whole integer where *m* is the total number of features) was the optimal number to select when studying binary classification with imbalanced data corpora.

Finally, preprocessing feature selection techniques are typically applied on the cleaned training data. Khoshgoftaar et al. (2010) investigated how the accuracy of feature selection methods changed when features were ranked on the cleaned training data and when it was performed on a subset of the data using their cutoff. They found that prediction models yielded higher accuracy when feature selection was performed on subsets of the training corpus. Unlike Márquez-Vera et al. (2016), this study did not perform feature selection during cross-validation.

Given the limitations of prior work, the following topics were identified as in need of attention. First, a statistical framework is essential to evaluate and compare the impactful features identified by these techniques. Second, a subset of the training corpus should be used to rank features. And third, feature selection should be integrated during the cross-validation step. None of the prior studies noted above discuss developing a systematic consensus ranking scheme to assess the merit of each feature prior to model training. Consideration of all these factors may allow for the development of more robust and interpretable pipelines that incorporate preprocessing feature selection techniques.

## Integration of feature selection with cross-validation

**Fig. 2:**
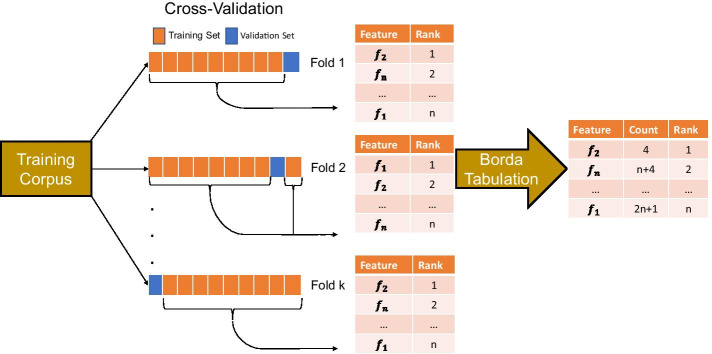
Overview of modified step where filter feature selection techniques rank features ${f}_{1},\dots ,{f}_{n}$ using Borda’s method

Figure 2 illustrates our methodology integrating feature selection with cross-validation and applying a consensus ranking methodology to identify pertinent predictors prior to model testing. After filter feature selection techniques tabulate the association between each feature and the dependent variable on each training fold, the sorted order of the importance for the *i*th feature can be considered as a permutation *P*_*j*_, *j* = 1,…*l* on *l* rankings with a linear weight assigned to each of the *l* positions in the permutation. The ranks, corresponding to the position of the features in ranked order can be aggregated across all folds and sorted to yield a final consensus.

The following steps are used to obtain a consensus ranking across all folds and feature rankings: (1) on each fold, sort the importance of the features in descending order based on values obtained from the filter feature selection techniques, (2) assign a ranking for each feature based on their importance in modeling the target outcome, (3) sum the individual rankings across all folds, and (4) sort the summed ranks to yield a final consensus ranking. A lower consensus ranking (i.e., closer to 1) indicates that the feature is ranked as highly important in the majority of cross-validation folds. This consensus ranking methodology, entitled Borda’s method, has been applied in political science to tabulate election results where the voting method is a scoring rule rather than a plurality (Borda, 1781; Fraenkel & Grofman, 2014; Reilly, 2002). To our knowledge, this methodology has not been incorporated into a comprehensive EDM pipeline.

To illustrate this in further detail, a tabular example is presented in Table 1 using eight features {A, B, C, D, E, F, G, H} and three folds. Borda’s method identifies features A and D as having the lowest Borda count in our training corpus. Based on the consensus rankings, a prespecified number of features can be chosen or a cutoff (e.g., Khoshgoftaar et al., 2007, 2010) can be applied. This method still works in the case of ties since all features whose Borda consensus ranking is less than a specified threshold (e.g., the four most important features) can be included in a final prediction model.

**Table 1:** Hypothetical example: Borda’s method with 8 features and three folds

Ranking position	Fold 1: Assigned ranking	Fold 2: Assigned ranking	Fold 3: Assigned ranking	Sorted consensus ranking	Consensus ranking position
1	A: 1	B: 1	D: 1	A: 6	1
2	C: 2	D: 2	A: 2	D: 6	1
3	D: 3	A: 3	B: 3	B: 11	3
4	G: 4	G: 4	F: 4	G: 13	4
5	F: 5	F: 5	G: 5	F: 14	5
6	H: 6	E: 6	E: 6	C: 17	6
7	B: 7	C: 7	H: 7	E: 20	7
8	E: 8	H: 8	C: 8	H: 21	8

## Application of modified pipeline to the science classroom

As an application, our investigation focused on implementing and evaluating the modified data science pipeline to forecast student performance in an introductory biology course at a public research institution in the United States. This classroom context was chosen because student success in this gateway baccalaureate science course is moderate for underrepresented minorities and first-generation college students at this institution and limits progress towards degree completion. Addressing this challenge is an institutional priority.

3225 students enrolled in this course over six semesters from fall 2014 to spring 2017 were studied (Table 2). The target outcome was each student’s transcript grade for the class categorized as a binary outcome: a failing course grade included marks of D^+^ and lower, while a passing course grade included marks of C^−^ and higher. A corpus of 57 university and course-specific features was obtained and amalgamated from the institution’s data warehouse. These features pertained to: (1) student demographics, (2) pre-college characteristics, (3) collegiate academic characteristics, (4) learning management system (LMS) logins, (5) financial aid metrics, and (6) biology course features. Summary statistics for the features can be found in Additional file 1: Material A.

**Table 2:** Summary grade statistics by term

Semester	Fail	Pass	Row total
Fall 2014 (n = 468)	93 (19.9%)	375 (80.1%)	468
Spring 2015 (n = 590)	44 (7.5%)	546 (92.5%)	590
Fall 2015 (n = 510)	116 (22.7%)	394 (77.3%)	510
Spring 2016 (n = 571)	24 (4.2%)	547 (95.8%)	571
Fall 2016 (n = 510)	74 (14.5%)	436 (85.5%)	510
Spring 2017 (n = 576)	27 (4.7%)	549 (95.3%)	576
Column total	378 (11.7%)	2847 (88.3%)	3225

**Fig. 3:**
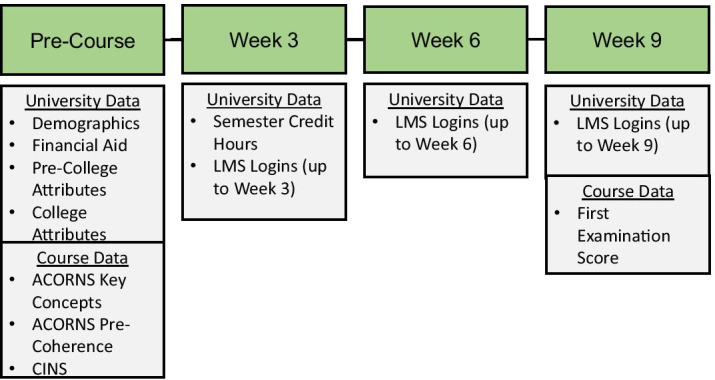
Features introduced at each time frame. Features are incrementally aggregated with those from prior time intervals

To investigate the differential predictive impact of the preprocessing feature selection techniques and DMMs at different time points in the course, models were constructed at pre-course, week 3, week 6, and week 9 (Fig. 3).

**Fig. 4:**
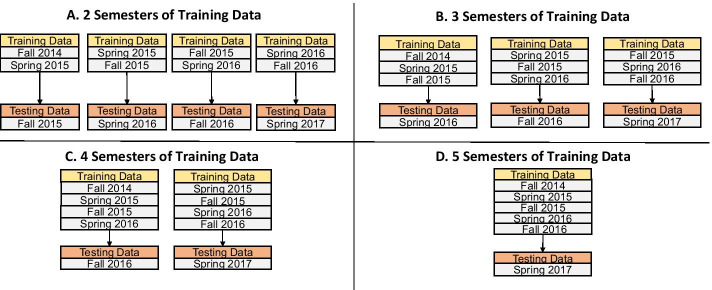
Prediction methodology overview. Modified from Bertolini et al. (2021)

To examine the stability of our methodology, the data pipeline was applied to forecast student performance in a single semester using two, three, four, and five prior semesters of training data (Fig. 4).

## Summary of modified data pipeline

**Fig. 5:**
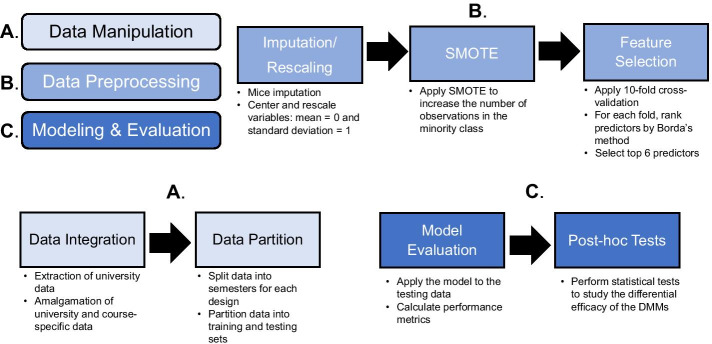
Modified data science pipeline. Modified from Bertolini et al. (2021)

Figure 5 depicts the modified data science pipeline for our study, which was implemented in R software (R Core Team, 2017). In summary, the data science pipeline encompassed the following eight steps. A comprehensive and detailed discussion of these pipeline steps can be found in Additional file 1: Material G.

1. University-specific and course-specific data are assembled.
2. The amalgamated corpus is divided into training and testing sets per Fig. 4.
3. Categorical features are converted into indicator variables.
4. Missing data are imputed using the predictive mean matching technique in R’s Multivariate Imputation by Chained Equations (MICE) package (Buuren & Groothuis-Oudshoorn, 2010).
5. Each feature is transformed to a z-score.
6. SMOTE is applied to the training data to address the class disparity.
7. Each DMM is trained using ten-fold cross-validation and applied to the testing data. When the four preprocessing filter feature selection techniques are applied, all features are ranked by Borda’s method and the ${\mathrm{log}}_{2}(m)$—cutoff was applied to select 57 = 5.83 ≈ 6 features to include in the final prediction model run on the testing corpus.
8. The model’s performance on the testing set is evaluated using the AUC metric for each DMM. Model performance omitting our pipeline revision is compared.

In the pipeline, we examined a collection of four filter feature selection techniques: CAE, Fisher’s scoring algorithm (FSA), IG, and relief attribute evaluation (RAE) to remove irrelevant features during preprocessing. IG and RAE were implemented in R via the FSelector package (Romanski & Kotthoff, 2013). A mathematical description of the preprocessing feature selection techniques can be found in Additional file 1: Material D.

Four common DMMs were also considered: logistic regression (LR), elastic net regression (GLMNET), random forest (RF), and extreme gradient boosting (XGBoost). All DMMs were implemented in R via the caret package (Kuhn, 2015). The caretList function was used to tune DMM hyperparameters using a “tuneLength” parameter of six.

### Statistical analysis

To address RQ 1, a heat map was used to visualize the mean percent difference in the AUC values between pipelines incorporating preprocessing feature selection techniques and our ranking methodology, with pipelines omitting them for all four time points during the term. The statistical significance of these methods was ascertained using a multiple regression model. Estimated coefficients and p-values can be found in Additional file 1: Material H.

For RQ 2, the SC metric introduced by Nogueira and Brown (2016) was computed for all training and testing corpora to assess the stability of the filter feature selection techniques. In our research context, the number of data subsets is 10, corresponding to each fold in ten-fold cross-validation. Analysis of variance (ANOVA) results can be found in Additional file 1: Material H.

For RQ 3, the Jaccard index was computed between each pair of preprocessing feature selection techniques to numerically quantify whether the top six features identified during the Borda tabulation were similar between techniques (Jaccard, 1901). A list of predictors identified as being most representative in modeling the target outcome, across all time frames and preprocessing feature selection techniques, was generated. ANOVA results can be found in Additional file 1: Material H.

## Results

**Fig. 6:**
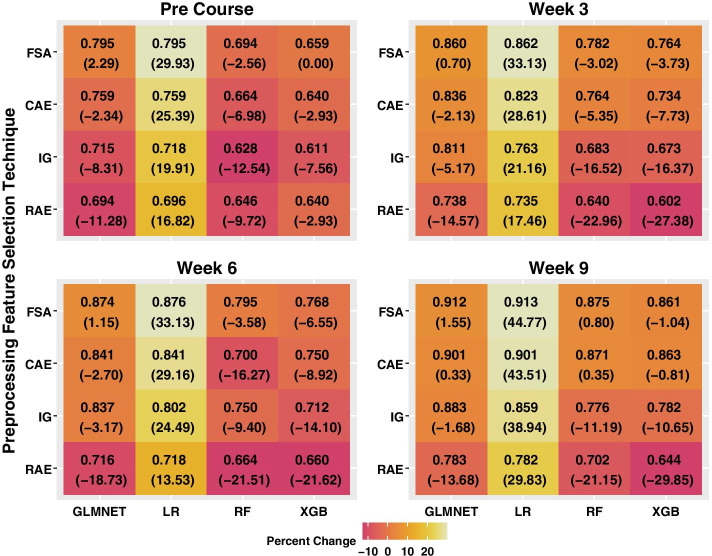
Average percent difference between the AUC values obtained using and omitting feature selection. In each data table entry, the first number denotes the mean AUC, across all training and testing corpora, when preprocessing feature selection are applied, and six features are used as input to the DMM. The entries in parenthesis correspond to the mean percent difference in the AUC, denoted by the colored palettes

*(RQ 1) Do preprocessing feature selection techniques enhance the predictive efficacy of DMMs compared to when this step is omitted from the EDM pipeline?* To examine the impact of the preprocessing feature selection techniques on the AUC metric, we tabulated the percent difference in the mean AUC when feature selection techniques were applied and when these methods were omitted for each training and testing corpus (Fig. 6). A positive percent difference, denoted in parentheses, indicated that using the six features identified by Borda’s method improved the mean AUC compared to when all features were used in the pipeline. A negative percent difference indicated that using the limited set of features decreased the mean AUC and did not enhance overall predictive efficacy.

On average, the AUC results obtained for LR, RF, and XGBoost were 0.055 (t-value = − 6.759, p-value < 0.0001, see Additional file 1: Material H), 0.073 (t-value = − 8.962, p-value < 0.0001, see Additional file 1: Material H) and 0.094 (t-value = − 11.523, p-value < 0.0001, see Additional file 1: Material H) points lower than GLMNET, respectively. Across all time frames and preprocessing feature selection techniques, the mean AUC values increased between 13.53 and 44.77% for LR. For this DMM, all percent differences for FSA exceeded 29.93%. By week 9, for the feature selection techniques CAE and FSA, the mean AUC values for LR exceeded 0.90 and achieved comparable performance with the ensemble method GLMNET. The largest improvement in the mean AUC for GLMNET occurred prior to course commencement using FSA (2.29%).

Compared to when preprocessing feature selection techniques were omitted from the data science pipeline, FSA and CAE significantly increased the AUC, on average, by 0.058 (t-value = 6.435, p-value < 0.0001, see Additional file 1: Material H) and 0.040 (t-value = 4.391, p-value < 0.0001, see Additional file 1: Material H) points, respectively. However, feature selection techniques reduced the mean AUC for RF and XGBoost across all techniques and time frames. During week 3, week 6, and week 9, the mean AUC values for RAE decreased between 21.15 and 29.85% for these ensemble DMMs. RAE was the worst performing preprocessing feature selection technique and yielded AUC values that were, on average, 0.068 points lower compared to when feature selection was omitted (t-value = − 7.501, p-value < 0.0001, see Additional file 1: H).

*(RQ 2) How consistent are the relevant features identified by the preprocessing feature selection techniques on subsets of the training data?* Ten training folds were used to evaluate feature relevancy prior to applying Borda’s method to select the top six features in the data pipeline. The SC metric was used to assess the stability between the feature selection methods and identify whether the features chosen were similar across cross-validation folds and corpora. Table 3 depicts the mean SC metric by testing data semester (spring or fall), and training corpus size. Across all time frames, the preprocessing features selection techniques CAE and FSA were the most stable with mean SC values ranging between 0.87 and 1.00. The SC values for these filter methods were not statistical different from one another (t-ratio = − 0.860, adjusted p-value = 0.825, see Additional file 1: Material H). The values for IG were moderately high and the lowest mean SC metric occurred for a fall testing semester at week 6 using two training corpora (mean SC: 0.73). However, RAE was highly unstable across all training and testing corpora. The highest mean SC metric was only 0.31 at week 9 using three training semesters. The estimated mean difference between the SC values between IG and RAE was 0.654 (t-ratio = 52.512, adjusted p-value < 0.0001, see Additional file 1: Material H).

**Table 3:** Mean SC metric across all spring and fall testing corpora and training corpora sizes

Testing corpus	Time frame	Feature selection technique	Number of training semesters			
			2	3	4	5
Spring	Pre	CAE	0.94	0.96	0.96	0.96
		FSA	0.94	0.95	0.87	1.00
		IG	0.86	0.92	0.93	0.81
		RAE	0.30	0.19	0.24	0.16
	Week 3	CAE	0.95	0.90	0.91	0.89
		FSA	0.96	0.95	1.00	0.90
		IG	0.82	0.89	0.88	0.74
		RAE	0.17	0.17	0.18	0.13
	Week 6	CAE	0.95	0.97	1.00	0.90
		FSA	0.92	0.94	0.89	0.94
		IG	0.91	0.85	0.85	0.79
		RAE	0.20	0.19	0.19	0.16
	Week 9	CAE	1.00	0.96	1.00	0.89
		FSA	0.96	1.00	1.00	1.00
		IG	0.93	0.88	0.88	0.81
		RAE	0.19	0.17	0.25	0.24
Fall	Pre	CAE	0.93	1.00	1.00	
		FSA	0.92	1.00	1.00	
		IG	0.86	0.85	0.86	
		RAE	0.19	0.23	0.25	
	Week 3	CAE	0.93	1.00	0.87	
		FSA	0.97	1.00	1.00	
		IG	0.79	0.86	0.88	
		RAE	0.23	0.13	0.17	
	Week 6	CAE	1.00	1.00	0.96	
		FSA	0.97	0.93	1.00	
		IG	0.73	0.76	0.85	
		RAE	0.16	0.14	0.21	
	Week 9	CAE	0.93	1.00	0.90	
		FSA	0.95	1.00	1.00	
		IG	0.83	0.96	0.80	
		RAE	0.16	0.31	0.15	

Based on our prediction design, no fall testing corpus was available when five training semesters were used

**Fig. 7:**
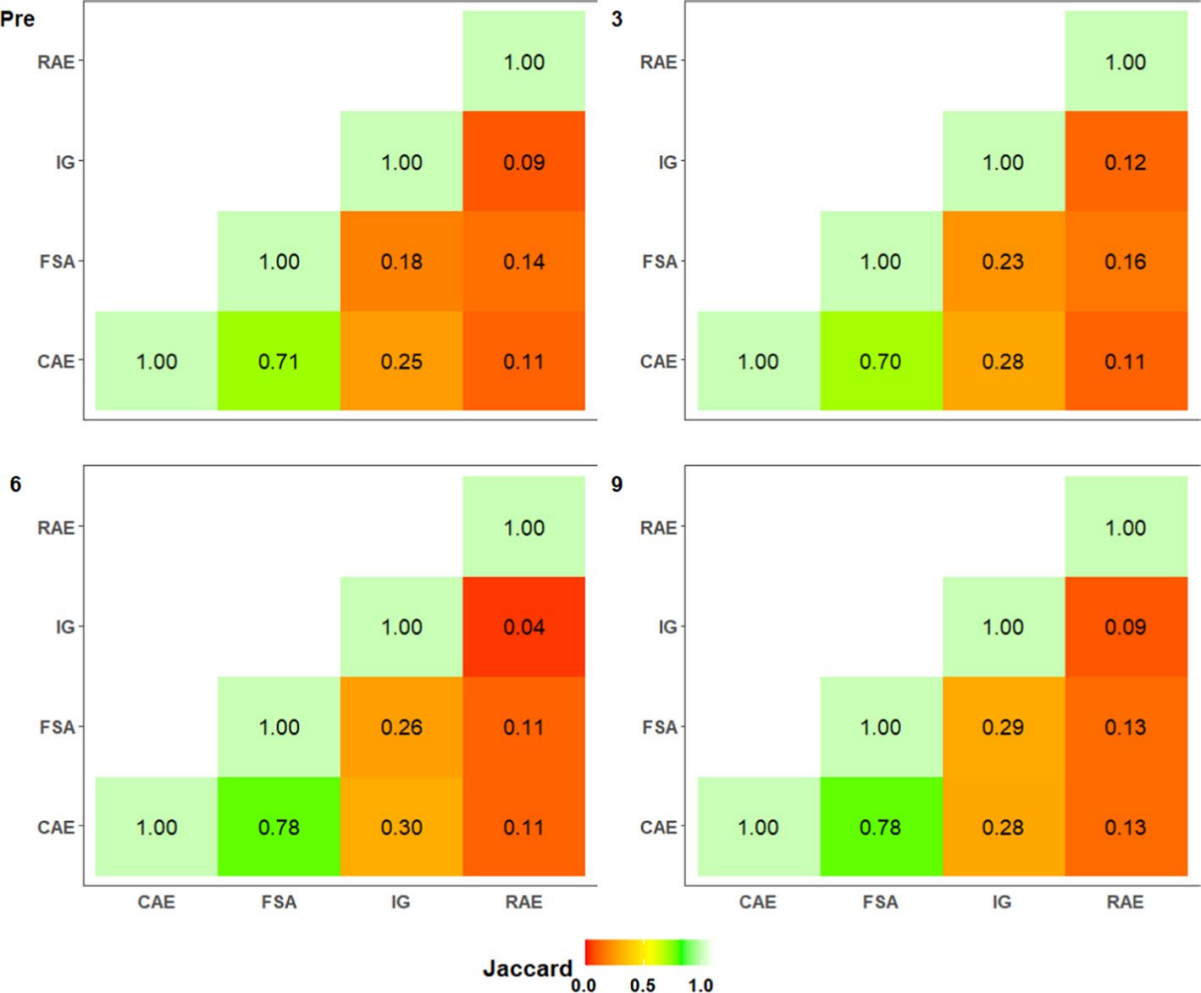
Mean Jaccard index correlation matrix for each time frame and preprocessing feature selection technique

*(RQ 3) What features do different feature selection techniques identify as contributing factors to student performance in the collegiate biology classroom, providing interpretable and actionable information for faculty and stakeholders?* The primary advantage of using preprocessing filter feature selection techniques is their ability to select and identify impactful features prior to model fitting in prediction pipelines. The Jaccard Index was tabulated for each pair of filter feature selection algorithms in order to identify whether the top six features (from Khoshgoftaar et al., 2007, 2010) used as input to the DMMs following the Borda tabulation were similar across all training and testing corpora (Fig. 7). CAE and FSA yielded the most similar set of the highest ranked features, as indicated by mean Jaccard indices ranging between 0.70 and 0.78, across all DMMs and training corpora sizes. CAE and RAE identified a divergent set of features, as indicated by mean Jaccard indices ranging between 0.11 and 0.13, respectively. The Jaccard indices between the filter methods CAE and FSA were significantly higher than all other pairs of filter methods (all t-ratios > 9.281, all adjusted p-values < 0.0001, see Additional file 1: Material H).

**Fig. 8:**
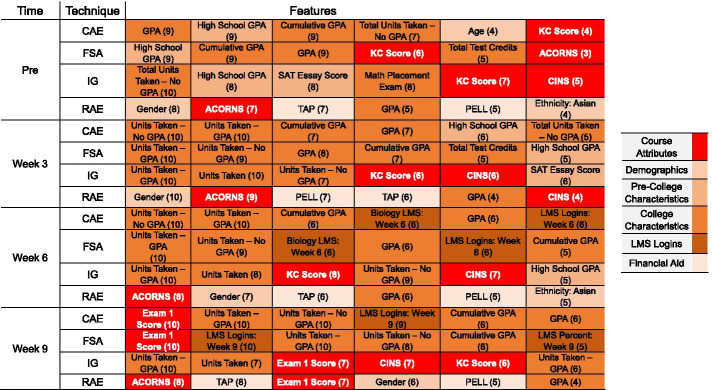
Top predictors identified across all times frames and feature selection techniques

Additional file 1: Material C provides detailed figures examining the top six features identified across all prediction scenarios and time frames. Figure 8 provides a tabular summary of the highest ranked features across all training corpora by time frame. The count entry was calculated by tabulating the number of times the predictor appeared in the top six features identified by Borda’s method across all training corpora. The maximum count total is 10. Pre-collegiate and collegiate academic features encompassed the majority of the highly impactful predictors. Student collegiate grade point average (GPA), high school GPA, and performance on the CI assessments were highly ranked at pre-course and continued to be important predictors in subsequent weeks of the term. At week 3 and week 6, the highest ranked features pertained to the number of credits the student was currently taking (the same semester they were enrolled in the biology course). Aside from the student’s gender, limited demographic characteristics were identified as being predictive of student classroom performance. While LMS logins were not the highest ranked features, they did appear in the Borda tabulation during the sixth week of the course. At week 9, the student’s first examination score was identified by all four filter techniques as being highly predictive of final course grade.

## Discussion

This study explored three research questions concerning the use of preprocessing feature selection techniques in the development of modified and interpretable data science pipelines to forecast student performance. We discuss the answers to our research questions, the broader implications of this work, and conclude with the limitations of this study.

The answer to RQ 1 is that the preprocessing feature selection techniques CAE and FSA significantly improved the mean AUC for the DMMs LR and GLMNET in our data pipeline for collegiate biology. In other EDM studies, CAE was identified as being the most advantageous filter technique due to its ability to assess the impact of each predictor by its association with the target outcome (Mangal & Holm, 2018). Moreover, applied research in other subjects has shown that the predictive performance of DMMs improves when CAE was applied prior to model fitting, compared to other filter methods including IG (Anuradha & Velmurugan, 2016; Doshi, 2014; Karegowda et al., 2010). In simulation studies on synthetic corpora, CAE was shown to be the most suitable filter preprocessing feature selection technique for omitting most non-representative features of the target outcome (Bolón-Canedo et al., 2013). However, several research studies have also found that CAE does not always yield an improvement in prediction performance. Osman et al. (2017) found that this technique only improved the predictive efficacy of DMMs in 25% of models and performed 33% worse than wrapper methods, while Zaffar et al. (2018) found that CAE achieved comparable performance with IG and RAE. Considerably less research has examined FSA since this technique can only be applied when the outcome variable is categorical (Aggarwal, 2014).

Despite this, there is a large quantity of research outside of EDM which found that other filter preprocessing feature selection techniques, including IG and RAE, are more successful in selecting highly important features than CAE and FSA (Herrera et al., 2002; Koprinska et al., 2015; Yang & Pedersen, 1997). Bolón-Canedo et al. (2013) noted IG’s positive performance in corpora with a copious number of non-representative features and a small ratio between the number of observations and the number of non-representative features. Furthermore, IG and RAE have also been shown to produce less accurate results when there is considerable variability and noise in the data corpus. For RAE, since this method is “intended as a screener to identify a subset of features that may not be the smallest,” it has the potential to identify redundant features as being highly predictive of the target outcome (Todorov, 2016, p. 96).

By using preprocessing filter techniques, instead of aggregating a large data corpus using all available features from stakeholders, researchers can extract a handful of relevant features from different educational records in order to develop robust and interpretable pipelines to guide educational administrators and faculty in designing interventions to improve student learning outcomes. Furthermore, in education, some researchers are hesitant to incorporate a large number of features and multiple data sources to bolster predictive models because they fear that errors in individual corpora will magnify when independent records are aggregated together (Bollier & Firestone, 2010; Strauss, 2016). We attribute the large amount of variability in the features assembled (see Additional file 1: Material A) as the likely reason for the overall poorer performance of IG and RAE across all DMMs and prediction methodologies in our modified pipeline.

The ensemble technique GLMNET significantly outperformed all other DMMs and improved the AUC metric when feature selection was applied as a preprocessing step to select six features. GLMNET is advantageous in binary classification due to its ability to select or exclude correlated covariates, and its use of regularization parameters to constrain the magnitudes of coefficients (Bertolini & Finch, in press; Jiménez et al., 2019; Kirpich et al., 2018; Lu & Petkova, 2014). However, it is important to note that some applied research found that this method performed worse than other ensemble techniques such as RF (Alexandro, 2018; Ransom et al., 2019).

The largest improvement in the AUC for feature selection was for the non-ensemble DMM LR. Traditionally, wrapper feature selection methods are commonly employed to improve predictive efficacy for LR (see Murtaugh, 1998; Harrell Jr, 2015). In our study, we demonstrated that preprocessing filter feature selection techniques also enhanced the performance of LR in achieving comparable AUC values with GLMNET and exceeded those of RF and XGBoost. While ensemble black-box techniques have been shown to enhance the performance of DMMs (Abdulazeez & Abdulwahab, 2018; Amrieh et al., 2016; Aulck et al., 2017; Beemer et al., 2018; Lisitsyna & Oreshin, 2019; Stapel et al., 2016), several EDM studies found that non-ensemble techniques performed better than ensemble models (Adekitan & Noma-Osaghae, 2019; Bucos & Drăgulescu, 2018). A limitation to these prior studies is that they neither incorporated preprocessing filter feature selection techniques nor utilized course-specific information in their analyses; rather, they focused on demographic and student academic achievements in their data pipelines.

We had anticipated that the ensemble techniques RF and XGBoost would be more competitive with the other DMMs. In particular, XGBoost has emerged as a prominent DMM and has won several data science competitions (Adam-Bourdarios et al., 2015; Nielsen, 2016). However, there are still a small number of studies where XGBoost performed worse than other ensemble algorithms (e.g., RF) for modeling a binary outcome (Chen et al., 2019; Gamie et al., 2019). We chose to apply the linear version of the XGBoost algorithm, ‘xgbLinear’, because it uses a similar linear penalty function as GLMNET. Other extreme gradient boosting techniques include the tree-based algorithms ‘xgbDART’ and ‘xgbTree’ in R’s caret package. For binary classification problems, ‘xgbTree’ and ‘xgbLinear’ have been shown to achieve comparable performance on standard machine learning data sets (Müller, 2018). Our research shows that filter methods have the potential to bolster the performance of non-ensemble DMMs and can be used for developing more robust and less convoluted educational data science pipelines, while making ensemble black-box methods more interpretable.

RQ 2 addressed how stable feature selection techniques are at selecting the most relevant features on subsamples of the training data. Our methodology incorporated feature selection with the cross-validation step of the data science pipeline and evaluated stability using the SC metric, a statistic that (to our knowledge) has not been applied in EDM. Researchers seek stable algorithms so that data pipelines can generalize to independent corpora and consistent features can be identified as pertinent in modeling the target outcome. The high stability of CAE and FSA in selecting similar sets of features during preprocessing indicate that these methods can identify the features associated with student performance in data science pipelines for collegiate biology. Highly unstable techniques across all training and testing corpora such as RAE demonstrate that this algorithm had difficulty identifying features representative of the target outcome, leading to biased predictions. Educational researchers are encouraged to try various techniques in order to achieve optimal performance when constructing data-driven tools until more findings are published on the stability of these algorithms.

The answer to RQ 3 is that academic (pre-collegiate and collegiate) attributes and course-specific features were found to be more highly predictive of student performance than demographic factors. This finding is consistent with other education research (Miller-Cotto & Schunn, 2020; Salehi et al., 2019; Simmons & Heckler, 2020; Thomas & Galambos, 2004). It was surprising that LMS logins were not identified as being highly relevant in the majority of our prediction models, considering the prominence of digital tools in the classroom environment. However, the impact of LMS logins in predicting student outcomes has been mixed in EDM. Some studies found them to be valuable for accurately predicting performance in online classroom settings (Al-Shabandar et al., 2017; Lisitsyna & Oreshin, 2019; Morris et al., 2005; Tan et al., 2019); however, poorer prediction performance was achieved for blended courses incorporating online learning and in-class instruction (Conijn et al., 2016). Since the biology course studied is a lecture-based in-person course where the LMS is used for instructor/student communication and for posting lecture notes, the course delivery method may be attributed to these predictors being less of a contributing factor to retention and attrition than in other studies. Since the introduction of online learning is becoming omnipresent in higher education, information and data acquired from these virtual tools can enhance the generality of student success predictions (Aljawarneh, 2020; Vovides et al., 2007). Given the recent shift to online instruction (Hodges et al., 2020), we are interested in extending our analysis to develop educational data pipelines to model student performance in these settings.

Communicating to faculty and other stakeholders how EDM pipelines and EWSs work is often challenging given the complexity of the corpora and associated analytical methods. The features responsible for DMM predictions can be murky, which limits understanding of how failure predictions should be addressed (e.g., academic vs. non-academic interventions). Indeed, the production of interpretable knowledge is an important consideration for these computational tools (Conati et al., 2018; Putnam & Conati, 2019), and data mining and ensemble learning have been criticized accordingly (Brooks et al., 2020; Elton, 2020; Jha et al., 2019). Interpretable pipelines that can offer a clearer understanding of the relationships between different features and their impact on model performance could help to generate understanding and formulate action among stakeholders (Arrieta et al., 2020; Rudin, 2019). Our modified pipeline allows for a comparative assessment of how specific feature subsets, identified by filter feature selection techniques during cross-validation, impact the sensitivity of DMMs in quantifying variability of student performance predictions in data pipelines.

Since instructors may be unsure how to utilize predictions of student performance in the classroom, working with a limited set of features identified prior to developing a prediction model is more feasible to address conceptual difficulties and facilitate a dialogue with academic stakeholders in taking a proactive approach to improve retention and aid struggling students. That way, supplemental instructional resources can be targeted for specific cohorts of students based on specific academic and/or non-academic attributes identified during data preprocessing to ensure high-risk students are targeted in receiving sufficient assistance and psychosocial support. More importantly, this methodology allows for the increased use of ensemble and “black-box” DMMs since educators have a better understanding of the student attributes that serve as inputs to these pipelines and their effect on prediction performance can be quantified in more direct ways. The interpretability of data pipelines and their underlying prediction algorithms in EDM will continue to play a major role in the application of computational tools that are used to infer student performance outcomes.

### Limitations

Dimensionality reduction techniques (e.g., principal component analysis (Van der Maaten et al., 2009), autoencoders (Hinton & Salakhutdinov, 2006), and random projection (Bingham & Mannila, 2001) were not considered in this study despite their popularity in fields outside of EDM. Unlike feature selection, these techniques provide limited interpretability due to altering the original representation and scale of the data (Alpaydin, 2020; Mangal & Holm, 2018). A more detailed study comparing the predictive efficacy of DMMs when feature selection and dimensionality reduction techniques are applied as a preprocessing step in our modified data science pipeline can be performed in future studies.

Wrapper and embedded feature selection methods were not considered despite their popularity in the data-enabled sciences. While other studies have examined these techniques (see Chandrashekar & Sahin, 2014; Mangal & Holm, 2018), we chose to focus on filter methods since they are independent of the DMM. A more comprehensive study examining the predictive efficacy of wrapper and embedded techniques would be a pragmatic next step.

In our prediction models, we chose to have the statistical software pick the hyperparameters for each DMM during training using a tuneLength value of six from the caret package in R. This may have contributed to the overall poorer prediction performance of RF and XGBoost. Examining a comprehensive grid search of tuning parameters, as well as focusing on other libraries and programming languages that implement these DMMs and preprocessing feature selection techniques, may enhance pipeline performance (Bertolini and Finch, in press). Furthermore, our feature selection methodology only examined a single filter cutoff (Khoshgoftaar et al., 2007, 2010), and did not consider exploring other cutoffs examined in feature selection literature (Belanche & González, 2011; Bolón-Canedo et al., 2013; Breiman et al., 1984). These alternative cutoffs can be examined in future studies.

Aside from the student’s first examination score, the other features incorporated into the data pipeline at weeks three, six, and nine did not include course assessments, CIs, nor standardized instruments that directly measured student comprehension, memory retention of course topics, and concept mastery in the biology course. While students exhibit documented heterogeneous longitudinal learning patterns in large introductory collegiate science courses (see Sayre & Heckler, 2009; Nehm et al., 2012; Wang, 2018), the premise of this study centered on enhancing educational data pipelines and therefore, we only included a limited set of course-specific features in the corpora. While not considered, the utility of other information extracted from LMSs aside from student login data (e.g., access to course deliverables) can be examined to further study student comprehension, learning, and course interaction throughout the duration of the class. Future studies should actively work to incorporate diverse course-specific data types quantifying memory retention into prediction pipeline studies.

A final limitation of this study is the application of this modified pipeline to a single collegiate biology class. Since the performance of preprocessing feature selection techniques and DMMs differ based on the application and corpora examined, similar trends in student performance predictions for these algorithms may differ in other educational contexts (e.g., flipped classroom environment, quarter-long courses) and courses (e.g., introductory chemistry, physics, and mathematics courses). We are currently conducting a comprehensive simulation study examining the robustness of this modified pipeline to discern variability in its prediction performance. By using synthetic data in these studies, the results obtained would not be dependent on the underlying corpus and research context.

## Conclusion

Our work introduced a systematic ranking system capable of identifying features associated with a target outcome during preprocessing. This step can potentially simplify traditional data pipelines, make ensemble DMMs more interpretable, and allow researchers to examine highly impactful features prior to training prediction models. In an application to the collegiate biology classroom, this pipeline step improved predictive efficacy for the DMMs LR and GLMNET, compared to when all features were used. The features identified on training folds were stable, consistent across different corpora, and provided insight into the academic factors contributing to retention and attrition. By precisely pinpointing the features that directly contribute to student performance, educational researchers can use feature selection and the modified pipeline devised to develop and deploy targeted interventions to help improve the academic success of undergraduate students. Future work is exploring how the complexity of predictive tools impact the ways faculty make sense of and use these predictions in their classrooms.

## Supplementary Information

**Additional file 1.** Material A: Summary statistics for features. Material B: AUC results for each preprocessing feature selection technique and DMM across all time frames, corpora sizes, and training and testing corpora. Material C: Top six ranked predictors selected by each preprocessing technique across all training corpora. Material D: Description of preprocessing feature selection techniques. Material E: Concept inventory assessments. Material F: Overview of data pipeline steps. Material G: Data pipeline for the collegiate biology classroom. Material H: AUC regression model & ANOVA analysis for SC metric and Jaccard index. Material I. Additional Materials References.

## Data Availability

All data analyzed in this study are available from the corresponding author on reasonable request.

## Abbreviations

ANOVA: Analysis of variance
AUC: Area under the curve
CAE: Correlation attribute evaluation
DMM: Data mining method
EDM: Educational data mining
EWS: Early warning system
FSA: Fisher’s scoring algorithm
GLMNET: Elastic net regression
GPA: Grade point average
IG: Information gain
LMS: Learning management system
LR: Logistic regression
MICE: Multivariate imputation by chained equations
RAE: Relief attribute evaluation
RF: Random forest
SMOTE: Synthetic minority oversampling technique
XGBoost: Extreme gradient boosting
