# Supplementary material for: Enhancing data pipelines for forecasting student performance: integrating feature selection with cross-validation
Source: Int J Educ Technol High Educ. 2021 Aug 17;18(1):44. doi: 10.1186/s41239-021-00279-6 (PMC8591701; doi:10.1186/s41239-021-00279-6)
Supplement: Supplementary file 1 — Additional file 1. Material A: Summary statistics for features. Material B: AUC results for each preprocessing feature selection technique and DMM across all time frames, corpora sizes, and training and testing corpora. Material C: Top six ranked predictors selected by each preprocessing technique across all training corpora. Material D: Description of preprocessing feature selection techniques. Material E: Concept inventory assessments. Material F: Overview of data pipeline steps. Material G: Data pipeline for the collegiate biology classroom. Material H: AUC regression model & ANOVA analysis for SC metric and Jaccard index. Material I. Additional Materials References. [file 41239_2021_279_MOESM1_ESM.docx]

**Additional file: Materials**

**Table of Contents**

A: Summary statistics for features5

Table S1: Descriptive statistics for categorical features by pass and fail status5

Table S2: Descriptive statistics for continuous features by pass and fail status6

Table S3: List of features introduced at each time frame8

B: AUC results for each preprocessing feature selection technique and DMM across all time frames, corpora sizes, and training and testing corpora 9

Table S4: Point estimate AUC results for fall 2015 and spring 2016 testing semester across all time frames, DMMs, and corpora sizes without preprocessing feature selection9

Table S5: Point estimate AUC results for fall 2016 and spring 2017 testing semester across all time frames, DMMs, and corpora sizes without preprocessing feature selection10

Table S6: Point estimate AUC results for fall 2015 and spring 2016 testing semester across all time frames, DMMs, and corpora sizes with Correlation Attribute Evaluation11

Table S7: Point estimate AUC results for fall 2016 and spring 2017 testing semester across all time frames, DMMs, and corpora sizes with Correlation Attribute Evaluation12

Table S8: Point estimate AUC results for fall 2015 and spring 2016 testing semester across all time frames, DMMs, and corpora sizes with Fisher’s Scoring Algorithm13

Table S9: Point estimate AUC results for fall 2016 and spring 2017 testing semester across all time frames, DMMs, and corpora sizes with Fisher’s Scoring Algorithm14

Table S10: Point estimate AUC results for fall 2015 and spring 2016 testing semester across all time frames, DMMs, and corpora sizes with Information Gain Attribute Evaluation15

Table S11: Point estimate AUC results for fall 2016 and spring 2017 testing semester across all time frames, DMMs, and corpora sizes with Information Gain Attribute Evaluation16

Table S12: Point estimate AUC results for fall 2015 and spring 2016 testing semester across all time frames, DMMs, and corpora sizes with Relief Attribute Evaluation17

Table S13: Point estimate AUC results for fall 2016 and spring 2017 testing semester across all time frames, DMMs, and corpora sizes with Relief Attribute Evaluation18

Table S14: SC metric for fall 2015 and spring 2016 testing corpus19

Table S15: SC metric for fall 2016 and spring 2017 testing corpus20

C: Top six ranked predictors selected by each preprocessing technique across all training corpora21

Figure S1: Top six ranked predictors: two training semesters and fall 2015 testing semester – pre-course21

Figure S2: Top six ranked predictors: two training semesters and fall 2015 testing semester – week 321

Figure S3: Top six ranked predictors: two training semesters and fall 2015 testing semester – week 622

Figure S4: Top six ranked predictors: two training semesters and fall 2015 testing semester – week 922

Figure S5: Top six ranked predictors: two training semesters and spring 2016 testing semester – pre-course23

Figure S6: Top six ranked predictors: two training semesters and spring 2016 testing semester – week 323

Figure S7: Top six ranked predictors: two training semesters and spring 2016 testing semester – week 624

Figure S8: Top six ranked predictors: two training semesters and spring 2016 testing semester – week 924

Figure S9: Top six ranked predictors: two training semesters and fall 2016 testing semester – pre-course25

Figure S10: Top six ranked predictors: two training semesters and fall 2016 testing semester – week 325

Figure S11: Top six ranked predictors: two training semesters and fall 2016 testing semester – week 626

Figure S12: Top six ranked predictors: two training semesters and fall 2016 testing semester – week 926

Figure S13: Top six ranked predictors: two training semesters and spring 2017 testing semester – pre-course27

Figure S14: Top six ranked predictors: two training semesters and spring 2017 testing semester – week 327

Figure S15: Top six ranked predictors: two training semesters and spring 2017 testing semester – week 628

Figure S16: Top six ranked predictors: two training semesters and spring 2017 testing semester – week 928

Figure S17: Top six ranked predictors: three training semesters and spring 2016 testing semester – pre-course29

Figure S18: Top six ranked predictors: three training semesters and spring 2016 testing semester – week 329

Figure S19: Top six ranked predictors: three training semesters and spring 2016 testing semester – week 630

Figure S20: Top six ranked predictors: three training semesters and spring 2016 testing semester – week 930

Figure S21: Top six ranked predictors: three training semesters and fall 2016 testing semester – pre-course31

Figure S22: Top six ranked predictors: three training semesters and fall 2016 testing semester – week 331

Figure S23: Top six ranked predictors: three training semesters and fall 2016 testing semester – week 632

Figure S24: Top six ranked predictors: three training semesters and fall 2016 testing semester – week 932

Figure S25: Top six ranked predictors: three training semesters and spring 2017 testing semester – pre-course33

Figure S26: Top six ranked predictors: three training semesters and spring 2017 testing semester – week 333

Figure S27: Top six ranked predictors: three training semesters and spring 2017 testing semester – week 634

Figure S28: Top six ranked predictors: three training semesters and spring 2017 testing semester – week 934

Figure S29: Top six ranked predictors: four training semesters and fall 2016 testing semester – pre-course35

Figure S30: Top six ranked predictors: four training semesters and fall 2016 testing semester – week 335

Figure S31: Top six ranked predictors: four training semesters and fall 2016 testing semester – week 636

Figure S32: Top six ranked predictors: four training semesters and fall 2016 testing semester – week 936

Figure S33: Top six ranked predictors: four training semesters and spring 2017 testing semester – pre-course37

Figure S34: Top six ranked predictors: four training semesters and spring 2017 testing semester – week 337

Figure S35: Top six ranked predictors: four training semesters and spring 2017 testing semester – week 638

Figure S36: Top six ranked predictors: four training semesters and spring 2017 testing semester – week 938

Figure S37: Top six ranked predictors: five training semesters and spring 2017 testing semester – pre-course39

Figure S38 Top six ranked predictors: five training semesters and spring 2017 testing semester – week 339

Figure S39: Top six ranked predictors: five training semesters and spring 2017 testing semester – week 640

Figure S40: Top six ranked predictors: five training semesters and spring 2017 testing semester – week 940

D: Description of preprocessing feature selection techniques41

Correlation Attribute Evaluation (CAE)41

Fisher’s Scoring Algorithm (FSA)41

Information Gain Attribute Evaluation (IG)41

Relief Attribute Evaluation (RAE)42

E: Concept inventory assessments43

Assessing Contextual Reasoning about Natural Selection (ACORNS)43

Conceptual Inventory of Natural Selection (CINS)43

F: Overview of data pipeline steps44

Step one: data manipulation44

Step two: data preprocessing44

Step three: data modeling44

Step four: model evaluation45

G: Data pipeline for the collegiate biology classroom46

Step one: data manipulation46

Step two: data preprocessing46

Step three: data modeling47

Step four: model evaluation47

H: AUC regression model & ANOVA analysis for SC metric and Jaccard index49

Table S16: Regression coefficient estimates and significance tests for the effect of (1) testing semester, (2) number of training semesters, (3) time frame, (4) data mining method, and (5) preprocessing feature selection technique.50

Table S17: Tukey’s honestly significant difference multiple comparisons analysis for the SC metric between each pair of preprocessing feature selection techniques51

Table S18: Tukey’s honestly significant difference multiple comparisons analysis for the Jaccard index between each pair of preprocessing feature selection techniques52

I: Additional Materials References53

**A: Summary statistics for features**

Table S1: Descriptive statistics for categorical features by pass and fail status

| Feature | Total | Missing | Factor | Fail | Pass |
| --- | --- | --- | --- | --- | --- |
| Academic Program | 3225 | 33 (1.0%) | Arts & Sciences | 287 | 2314 |
|  |  |  | Engineering & Applied Sciences | 51 | 218 |
|  |  |  | Other | 35 | 287 |
| Academic Load | 3225 | 0 (0.0%) | Part Time | 21 | 80 |
|  |  |  | Full Time | 357 | 2767 |
| Academic Level | 3225 | 0 (0.0%) | Freshmen | 72 | 295 |
|  |  |  | Sophomore | 149 | 1052 |
|  |  |  | Junior | 88 | 967 |
|  |  |  | Senior | 62 | 508 |
|  |  |  | Graduate Student | 7 | 25 |
| Campus Residence | 3225 | 322 (0.0%) | Commuter | 73 | 354 |
|  |  |  | Off Campus | 14 | 152 |
|  |  |  | On Campus | 264 | 2046 |
| Citizenship | 3225 | 33 (1.0%) | Alien | 108 | 396 |
|  |  |  | Native | 241 | 2239 |
|  |  |  | Naturalized | 24 | 184 |
| Citizenship Country | 3225 | 0 (0.0%) | USA | 241 | 2240 |
|  |  |  | Outside USA | 137 | 607 |
| Enrollment Status | 3225 | 33 (1.0%) | Continuing Student | 304 | 2496 |
|  |  |  | Graduate Student | 7 | 25 |
|  |  |  | New Freshman | 28 | 121 |
|  |  |  | New Transfer | 34 | 177 |
| Ethnicity | 3225 | 0 (0.0%) | Asian | 168 | 1139 |
|  |  |  | Black | 24 | 198 |
|  |  |  | Hispanic | 47 | 296 |
|  |  |  | White | 115 | 988 |
|  |  |  | Other | 24 | 226 |
| First Term Indicator | 3225 | 0 (0.0%) | First Term | 60 | 290 |
|  |  |  | Not First Term | 318 | 2557 |
| Gender | 3225 | 0 (0.0%) | Female | 169 | 1561 |
|  |  |  | Male | 209 | 1286 |
| Income Level | 3225 | 0 (0.0%) | < $29,999 | 34 | 205 |
|  |  |  | $30,000 - $84,999 | 43 | 223 |
|  |  |  | > $85,000 | 21 | 120 |
|  |  |  | Not Applicable unreported / | 280 | 2299 |
| Program Participant | 3225 | 0 (0.0%) | Yes | 19 | 80 |
|  |  |  | No | 359 | 2767 |
| Pell Grant Indicator | 3225 | 825 (25.6%) | Yes | 128 | 989 |
|  |  |  | No | 86 | 1197 |
| Residency State | 3225 | 0 (0.0%) | State where institution is located | 273 | 2409 |
|  |  |  | Another state | 105 | 438 |
| Tuition Assistance Recipient | 3225 | 941 (29.2%) | Yes | 118 | 1137 |
|  |  |  | No | 96 | 1033 |

Table S2: Descriptive statistics for continuous features by pass and fail status

| Feature | Total | | Missing Entries Total | Pass | Sample Size | | Mean | Median | | Standard Deviation | | Min | Max |
| --- | --- | --- | --- | --- | --- | --- | --- | --- | --- | --- | --- | --- | --- |
|  |  |  |  | Fail |  |  |  |  |  |  |  |  |  |
| Demographics | | | | | | | | | | | | | |
| Age | 3225 | | 33 (1.0%) | 2847 (88.3%) | 2819 (99.0%) | | 19.71 | 19.00 | | 2.02 | | 15.00 | 46.00 |
|  |  |  |  | 378 (11.7%) | 373 (98.7%) | | 20.38 | 20.00 | | 2.37 | | 17.00 | 35.00 |
| Pre-College Characteristics | | | | | | | | | | | | | |
| High School GPA | 3225 | | 311  (9.6%) | 2847 (88.3%) | 2598 (91.3%) | | 92.94 | 93.00 | | 4.72 | | 68.00 | 100.00 |
|  |  |  |  | 378 (11.7%) | 316 (83.6%) | | 88.88 | 89.50 | | 5.28 | | 66.00 | 99.00 |
| Math Placement Exam Score | 3225 | | 1062 (32.9%) | 2847 (88.3%) | 1911 (67.1%) | | 4.50 | 4.00 | | 1.83 | | 1.00 | 9.00 |
|  |  |  |  | 378 (11.7%) | 252 (66.7%) | | 3.70 | 3.00 | | 1.53 | | 1.00 | 9.00 |
| SAT 1600 Score | 3225 | | 508 (15.8%) | 2847 (88.3%) | 2465 (86.6%) | | 1264.00 | 1260.00 | | 135.22 | | 700.00 | 1600.00 |
|  |  |  |  | 378 (11.7%) | 252 (66.7%) | | 1200.78 | 1210.00 | | 146.28 | | 710.00 | 1530.00 |
| SAT Comp Score | 3225 | | 757 (23.5%) | 2847 (88.3%) | 2238 (78.6%) | | 1250.00 | 1250.00 | | 132.74 | | 700.00 | 1600.00 |
|  |  |  |  | 378 (11.7%) | 230 (60.8%) | | 1194.48 | 1210.00 | | 145.55 | | 710.00 | 1530.00 |
| SAT Essay Score | 3225 | | 876 (27.2%) | 2847 (88.3%) | 2129 (74.8%) | | 8.62 | 8.00 | | 1.22 | | 4.00 | 12.00 |
|  |  |  |  | 378 (11.7%) | 220 (58.2%) | | 8.22 | 8.00 | | 1.33 | | 4.00 | 12.00 |
| SAT Math Score | 3225 | | 757 (23.5%) | 2847 (88.3%) | 2238 (78.6%) | | 646.76 | 650.00 | | 78.02 | | 320.00 | 800.00 |
|  |  |  |  | 378 (11.7%) | 230 (60.8%) | | 627.30 | 640 | | 84.83 | | 310.00 | 800.00 |
| SAT MC Score | 3225 | | 876 (27.2%) | 2847 (88.3%) | 2129 (74.8%) | | 60.18 | 60.00 | | 8.50 | | 27.00 | 80.00 |
|  |  |  |  | 378 (11.7%) | 220 (58.2%) | | 56.45 | 57.00 | | 9.03 | | 23.00 | 80.00 |
| SAT Verbal Score | 3225 | | 757 (23.5%) | 2847 (88.3%) | 2238 (78.6%) | | 603.06 | 600.00 | | 79.79 | | 330.00 | 800.00 |
|  |  |  |  | 378 (11.7%) | 230 (60.8%) | | 567.17 | 570.00 | | 93.03 | | 340.00 | 800.00 |
| SAT Writing Score | 3225 | | 765 (23.7%) | 2847 (88.3%) | 2230 (78.3%) | | 605.45 | 610.00 | | 83.31 | | 310.00 | 800.00 |
|  |  |  |  | 378 (11.7%) | 230 (60.8%) | | 565.70 | 570.00 | | 84.99 | | 280.00 | 770.00 |
| College Academic Attributes | | | | | | | | | | | | | |
| Cumulative GPA previous semester | 3225 | | 468 (14.5%) | 2847 (88.3%) | 2464 (86.5%) | | 3.13 | 3.17 | | 0.55 | | 0.00 | 4.00 |
|  |  |  |  | 378 (11.7%) | 293 (77.5%) | | 2.56 | 2.56 | | 0.60 | | 0.55 | 4.00 |
| GPA previous semester | 3225 | | 468 (14.5%) | 2847 (88.3%) | 2464 (86.5%) | | 3.08 | 3.20 | | 0.70 | | 0.00 | 4.00 |
|  |  |  |  | 378 (11.7%) | 293 (77.5%) | | 2.36 | 2.48 | | 0.87 | | 0.00 | 4.00 |
| Total Cumulative previously | 3225 | | 468 (14.5%) | 2847 (88.3%) | 2464 (86.5%) | | 61.79 | 59.00 | | 28.99 | | 0.00 | 189.00 |
|  |  |  |  | 378 (11.7%) | 293 (77.5%) | | 54.39 | 47.00 | | 32.31 | | 6.00 | 162.50 |
| Total Grade Points previously | 3225 | | 468 (14.5%) | 2847 (88.3%) | 2464 (86.5%) | | 127.98 | 115.34 | | 82.73 | | 0.00 | 568.05 |
|  |  |  |  | 378 (11.7%) | 293 (77.5%) | | 97.32 | 74.33 | | 75.61 | | 6.00 | 370.62 |
| Total Test Credits previous | 3225 | | 468 (14.5%) | 2847 (88.3%) | 2464 (86.5%) | | 9.74 | 7.00 | | 9.67 | | 0.00 | 31.00 |
|  |  |  |  | 378 (11.7%) | 293 (77.5%) | | 3.55 | 0.00 | | 6.63 | | 0.00 | 30.00 |
| Total Units Passed no GPA previously | 3225 | | 468 (14.5%) | 2847 (88.3%) | 2464 (86.5%) | | 2.04 | 1.00 | | 2.51 | | 0.00 | 23.00 |
|  |  |  |  | 378 (11.7%) | 293 (77.5%) | | 1.95 | 1.00 | | 2.24 | | 0.00 | 15.00 |
| Total Units Passed – GPA previously | 3225 | | 468 (14.5%) | 2847 (88.3%) | 2464 (86.5%) | | 39.36 | 38.00 | | 24.48 | | 0.00 | 149.00 |
|  |  |  |  | 378 (11.7%) | 293 (77.5%) | | 33.49 | 26.00 | | 24.69 | | 3.00 | 114.00 |
| Total Units Passed previously | 3225 | | 468 (14.5%) | 2847 (88.3%) | 2464 (86.5%) | | 41.40 | 40.00 | | 25.61 | | 0.00 | 163.00 |
|  |  |  |  | 378 (11.7%) | 293 (77.5%) | | 35.44 | 28.00 | | 25.45 | | 3.00 | 117.00 |
| Total Units Taken for GPA previously | 3225 | | 468 (14.5%) | 2847 (88.3%) | 2464 (86.5%) | | 39.36 | 38.00 | | 24.48 | | 0.00 | 149.00 |
|  |  |  |  | 378 (11.7%) | 293 (77.5%) | | 37.75 | 29.00 | | 27.48 | | 3.00 | 140.00 |
| Total Units Taken in Progress previously | 3225 | | 468 (14.5%) | 2847 (88.3%) | 2464 (86.5%) | | 44.48 | 44.00 | | 27.63 | | 3.00 | 178.00 |
|  |  |  |  | 378 (11.7%) | 293 (77.5%) | | 44.22 | 32.00 | | 30.48 | | 12.00 | 187.00 |
| Total Units Taken no GPA previously | 3225 | | 468 (14.5%) | 2847 (88.3%) | 2464 (86.5%) | | 3.68 | 2.00 | | 4.67 | | 0.00 | 53.00 |
|  |  |  |  | 378 (11.7%) | 293 (77.5%) | | 6.47 | 4.00 | | 6.53 | | 0.00 | 48.00 |
| Total Units Transfer previously | 3225 | | 468 (14.5%) | 2847 (88.3%) | 2464 (86.5%) | | 10.64 | 0.00 | | 20.10 | | 0.00 | 120.00 |
|  |  |  |  | 378 (11.7%) | 293 (77.5%) | | 15.40 | 0.00 | | 24.65 | | 0.00 | 91.00 |
| Units Taken in Progress | 3225 | | 0  (0.0%) | 2847 (88.3%) | 2847 (100.0%) | | 15.30 | 15.00 | | 2.35 | | 2.00 | 23.00 |
|  |  |  |  | 378 (11.7%) | 378 (100.0%) | | 14.52 | 15.00 | | 2.57 | | 3.00 | 23.00 |
| Units Taken for GPA | 3225 | | 0  (0.0%) | 2847 (88.3%) | 2847 (100.0%) | | 14.46 | 15.00 | | 2.44 | | 2.00 | 23.00 |
|  |  |  |  | 378 (11.7%) | 378 (100.0%) | | 10.99 | 12.00 | | 4.53 | | 0.00 | 20.00 |
| Units Taken not for GPA | 3225 | | 0  (0.0%) | 2847 (88.3%) | 2847 (100.0%) | | 0.83 | 0.00 | | 1.48 | | 0.00 | 10.00 |
|  |  |  |  | 378 (11.7%) | 378 (100.0%) | | 3.52 | 3.00 | | 4.03 | | 0.00 | 19.00 |
| Units Test Credit | 3225 | | 0  (0.0%) | 2847 (88.3%) | 2847 (100.0%) | | 0.69 | 0.00 | | 3.54 | | 0.00 | 30.00 |
|  |  |  |  | 378 (11.7%) | 378 (100.0%) | | 0.27 | 0.00 | | 2.10 | | 0.00 | 26.00 |
| Units Transfer | 3225 | | 0  (0.0%) | 2847 (88.3%) | 2847 (100.0%) | | 3.68 | 0.00 | | 14.38 | | 0.00 | 120.50 |
|  |  |  |  | 378 (11.7%) | 378 (100.0%) | | 5.37 | 0.00 | | 17.01 | | 0.00 | 85.00 |
|  | |  |  |  |  |  | | |  | |  |  |  |
| Learning Management System (LMS) Logins | | | | | | | | | | | | | |
| Biology Course Logins Up to Week 3 | 3225 | | 4  (< 1%) | 2847 (88.3%) | 2847 (100.0%) | | 22.55 | 21.00 | | 11.31 | | 0.00 | 107.00 |
|  |  |  |  | 378 (11.7%) | 374 (98.9%) | | 16.94 | 16.00 | | 10.84 | | 0.00 | 72.00 |
| Biology Course Logins Up to Week 6 | 3225 | | 4  (< 1%) | 2847 (88.3%) | 2847 (100.0%) | | 28.55 | 27.00 | | 14.07 | | 0.00 | 194.00 |
|  |  |  |  | 378 (11.7%) | 374 (98.9%) | | 19.56 | 18.00 | | 12.57 | | 0.00 | 105.00 |
| Biology Course Logins Up to Week 9 | 3225 | | 4  (< 1%) | 2847 (88.3%) | 2847 (100.0%) | | 51.10 | 49.00 | | 23.77 | | 1.00 | 295.00 |
|  |  |  |  | 378 (11.7%) | 374 (98.9%) | | 36.49 | 35.00 | | 21.44 | | 1.00 | 177.00 |
| LMS Per Couse Logins Up to Week 3 | 3225 | | 7  (< 1%) | 2847 (88.3%) | 2847 (100.0%) | | 13.81 | 12.67 | | 6.95 | | 0.00 | 71.00 |
|  |  |  |  | 378 (11.7%) | 371 (98.1%) | | 11.32 | 10.67 | | 6.39 | | 0.00 | 44.00 |
| LMS Per Couse Logins Up to Week 6 | 3225 | | 7  (< 1%) | 2847 (88.3%) | 2847 (100.0%) | | 30.14 | 27.80 | | 14.11 | | 0.17 | 169.33 |
|  |  |  |  | 378 (11.7%) | 371 (98.1%) | | 23.63 | 21.80 | | 12.07 | | 1.67 | 74.40 |
| LMS Per Couse Logins Up to Week 9 | 3225 | | 7  (< 1%) | 2847 (88.3%) | 2847 (100.0%) | | 78.24 | 72.56 | | 36.07 | | 6.67 | 379.00 |
|  |  |  |  | 378 (11.7%) | 371 (98.1%) | | 52.64 | 48.80 | | 29.09 | | 3.17 | 169.4 |
| LMS Logins Up to Week 3 | 3225 | | 6  (< 1%) | 2847 (88.3%) | 2847 (100.0%) | | 74.55 | 69.00 | | 37.99 | | 0.00 | 308.00 |
|  |  |  |  | 378 (11.7%) | 372 (98.4%) | | 55.94 | 51.00 | | 32.56 | | 0.00 | 187.00 |
| LMS Logins Up to Week 6 | 3225 | | 7  (< 1%) | 2847 (88.3%) | 2847 (100.0%) | | 162.44 | 150.00 | | 75.99 | | 1.00 | 625.00 |
|  |  |  |  | 378 (11.7%) | 371 (98.1%) | | 117.36 | 106.00 | | 63.18 | | 10.00 | 380.00 |
| LMS Logins Up to Week 9 | 3225 | | 4  (< 1%) | 2847 (88.3%) | 2847 (100.0%) | | 421.76 | 391.00 | | 194.33 | | 14.00 | 1641.00 |
|  |  |  |  | 378 (11.7%) | 374 (98.9%) | | 261.99 | 234.50 | | 156.08 | | 6.00 | 929.00 |
| LMS Total Enrolled Courses | 3225 | | 7  (< 1%) | 2847 (88.3%) | 2847 (100.0%) | | 5.47 | 5.00 | | 1.10 | | 1.00 | 11.00 |
|  |  |  |  | 378 (11.7%) | 371 (98.1%) | | 5.01 | 5.00 | | 1.13 | | 1.00 | 9.00 |
| Financial Aid | | | | | | | | | | | | | |
| Disbursed Amount of Financial Aid | 3225 | | 825 (25.6%) | 2847 (88.3%) | 2186 (76.8%) | | 12381.69 | 11439.00 | | 7727.71 | | 0.00 | 50880.60 |
|  |  |  |  | 378 (11.7%) | 214 (56.6%)) | | 12347.63 | 11347.75 | | 7706.77 | | 0.00 | 40029.00 |
| Course – Specific Data | | | | | | | | | | | | | |
| ACORNS pre-course score | 3225 | | 1,131 (35.1%) | 2847 (88.3%) | 1995 (70.1%) | | 2.66 | 2.00 | | 1.69 | | 1.00 | 5.00 |
|  |  |  |  | 378 (11.7%) | 99 (26.2%) | | 3.37 | 5.00 | | 1.75 | | 1.00 | 5.00 |
| CINS pre-course score | 3225 | | 1,107 (34.3%) | 2847 (88.3%) | 1990 (69.9%) | | 10.51 | 10.00 | | 4.81 | | 0.00 | 20.00 |
|  |  |  |  | 378 (11.7%) | 128 (33.9%) | | 7.74 | 8.00 | | 4.32 | | 0.00 | 20.00 |
| Exam 1 Score | 3225 | | 79 (2.4%) | 2847 (88.3%) | 2817 (99.0%) | | 75.00 | 76.00 | | 13.00 | | 0.00 | 100.00 |
|  |  |  |  | 378 (11.7%) | 329 (87.0%) | | 54.00 | 53.00 | | 16.00 | | 0.00 | 91.00 |
| Total KC pre-course score | 3225 | | 1,131 (35.1%) | 2847 (88.3%) | 1995 (70.1%) | | 2.52 | 2.00 | | 1.82 | | 0.00 | 8.00 |
|  |  |  |  | 378 (11.7%) | 99 (26.2%) | | 1.67 | 1.00 | | 1.49 | | 0.00 | 7.00 |

Table S3: List of features introduced at each time frame

| Category | Features | Pre | 3 | 6 | 9 |
| --- | --- | --- | --- | --- | --- |
| Demographics | Age | ✓ | ✓ | ✓ | ✓ |
|  | Citizenship | ✓ | ✓ | ✓ | ✓ |
|  | Citizenship Country | ✓ | ✓ | ✓ | ✓ |
|  | Ethnicity | ✓ | ✓ | ✓ | ✓ |
|  | Gender | ✓ | ✓ | ✓ | ✓ |
|  | Income Level | ✓ | ✓ | ✓ | ✓ |
|  | Residency State | ✓ | ✓ | ✓ | ✓ |
| Pre-College Characteristics | High School GPA | ✓ | ✓ | ✓ | ✓ |
|  | SAT 1600 Score | ✓ | ✓ | ✓ | ✓ |
|  | SAT Comp Score | ✓ | ✓ | ✓ | ✓ |
|  | SAT Essay Score | ✓ | ✓ | ✓ | ✓ |
|  | SAT Math Score | ✓ | ✓ | ✓ | ✓ |
|  | SAT MC Score | ✓ | ✓ | ✓ | ✓ |
|  | SAT Verbal Score | ✓ | ✓ | ✓ | ✓ |
|  | SAT Writing Score | ✓ | ✓ | ✓ | ✓ |
| College Characteristics | Academic Level | ✓ | ✓ | ✓ | ✓ |
|  | Academic Load | ✓ | ✓ | ✓ | ✓ |
|  | Academic Program | ✓ | ✓ | ✓ | ✓ |
|  | Campus Residence | ✓ | ✓ | ✓ | ✓ |
|  | Cumulative Credits | ✓ | ✓ | ✓ | ✓ |
|  | Cumulative GPA | ✓ | ✓ | ✓ | ✓ |
|  | Enrollment Status | ✓ | ✓ | ✓ | ✓ |
|  | First Term Indicator | ✓ | ✓ | ✓ | ✓ |
|  | GPA | ✓ | ✓ | ✓ | ✓ |
|  | Math Placement Exam | ✓ | ✓ | ✓ | ✓ |
|  | Program Participant | ✓ | ✓ | ✓ | ✓ |
|  | Total Grade Points | ✓ | ✓ | ✓ | ✓ |
|  | Total Test Credits | ✓ | ✓ | ✓ | ✓ |
|  | Total Transfer Units | ✓ | ✓ | ✓ | ✓ |
|  | Total Units Passed | ✓ | ✓ | ✓ | ✓ |
|  | Total Units Passed: No GPA | ✓ | ✓ | ✓ | ✓ |
|  | Total Units Passed GPA | ✓ | ✓ | ✓ | ✓ |
|  | Total Units Taken | ✓ | ✓ | ✓ | ✓ |
|  | Total Units Taken – No GPA | ✓ | ✓ | ✓ | ✓ |
|  | Total Units Taken GPA | ✓ | ✓ | ✓ | ✓ |
|  | Units Taken – No GPA |  | ✓ | ✓ | ✓ |
|  | Units Taken - GPA |  | ✓ | ✓ | ✓ |
|  | Units Taken |  | ✓ | ✓ | ✓ |
|  | Units Test Credit |  | ✓ | ✓ | ✓ |
|  | Units Transfer |  | ✓ | ✓ | ✓ |
| LMS Logins | Biology LMS: Week 3 |  | ✓ |  |  |
|  | Biology LMS: Week 6 |  |  | ✓ |  |
|  | Biology LMS: Week 9 |  |  |  | ✓ |
|  | LMS Percent: Week 3 |  | ✓ |  |  |
|  | LMS Percent: Week 6 |  |  | ✓ |  |
|  | LMS Percent: Week 9 |  |  |  | ✓ |
|  | LMS Logins: Week 3 |  | ✓ |  |  |
|  | LMS Logins: Week 6 |  |  | ✓ |  |
|  | LMS Logins: Week 9 |  |  |  | ✓ |
|  | LMS Total Courses |  | ✓ | ✓ | ✓ |
| Financial Aid | Financial Aid Amount | ✓ | ✓ | ✓ | ✓ |
|  | Pell Grant Indicator | ✓ | ✓ | ✓ | ✓ |
|  | Tuition Assistance | ✓ | ✓ | ✓ | ✓ |
| Course Attributes | ACORNS | ✓ | ✓ | ✓ | ✓ |
|  | CINS | ✓ | ✓ | ✓ | ✓ |
|  | Exam 1 Score |  |  |  | ✓ |
|  | KC Score | ✓ | ✓ | ✓ | ✓ |

**B: AUC results for each preprocessing feature selection technique and DMM across all time frames, corpora sizes, and training and testing corpora**

Table S4: Point estimate AUC results for fall 2015 and spring 2016 testing semester across all time frames, DMMs, and corpora sizes without preprocessing feature selection

| Testing Data | Time Frame | Data Mining Method | Testing AUC: 2 semesters | Testing AUC: 3 semesters |
| --- | --- | --- | --- | --- |
| Fall 2015 | Pre | LR | 0.51 |  |
|  |  | GLMNET | 0.78 |  |
|  |  | RF | 0.75 |  |
|  |  | XGBoost | 0.75 |  |
|  | Week 3 | LR | 0.61 |  |
|  |  | GLMNET | 0.84 |  |
|  |  | RF | 0.80 |  |
|  |  | XGBoost | 0.81 |  |
|  | Week 6 | LR | 0.81 |  |
|  |  | GLMNET | 0.81 |  |
|  |  | RF | 0.81 |  |
|  |  | XGBoost | 0.82 |  |
|  | Week 9 | LR | 0.64 |  |
|  |  | GLMNET | 0.89 |  |
|  |  | RF | 0.87 |  |
|  |  | XGBoost | 0.89 |  |
| Spring 2016 | Pre | LR | 0.55 | 0.79 |
|  |  | GLMNET | 0.81 | 0.82 |
|  |  | RF | 0.79 | 0.77 |
|  |  | XGBoost | 0.77 | 0.76 |
|  | Week 3 | LR | 0.55 | 0.78 |
|  |  | GLMNET | 0.88 | 0.87 |
|  |  | RF | 0.85 | 0.82 |
|  |  | XGBoost | 0.83 | 0.76 |
|  | Week 6 | LR | 0.55 | 0.72 |
|  |  | GLMNET | 0.89 | 0.87 |
|  |  | RF | 0.86 | 0.84 |
|  |  | XGBoost | 0.86 | 0.81 |
|  | Week 9 | LR | 0.55 | 0.67 |
|  |  | GLMNET | 0.93 | 0.89 |
|  |  | RF | 0.89 | 0.86 |
|  |  | XGBoost | 0.88 | 0.86 |

Table S5: Point estimate AUC results for fall 2016 and spring 2017 testing semester across all time frames, DMMs, and corpora sizes without preprocessing feature selection

| Testing Data | Time Frame | Data Mining Method | Testing AUC: 2 semesters | Testing AUC: 3 semesters | Testing AUC: 4 semesters | Testing AUC: 5 semesters |
| --- | --- | --- | --- | --- | --- | --- |
| Fall 2016 | Pre | LR | 0.54 | 0.54 | 0.56 |  |
|  |  | GLMNET | 0.70 | 0.69 | 0.74 |  |
|  |  | RF | 0.76 | 0.73 | 0.75 |  |
|  |  | XGBoost | 0.72 | 0.68 | 0.72 |  |
|  | Week 3 | LR | 0.54 | 0.53 | 0.68 |  |
|  |  | GLMNET | 0.82 | 0.81 | 0.84 |  |
|  |  | RF | 0.82 | 0.81 | 0.83 |  |
|  |  | XGBoost | 0.82 | 0.80 | 0.80 |  |
|  | Week 6 | LR | 0.53 | 0.54 | 0.57 |  |
|  |  | GLMNET | 0.82 | 0.84 | 0.85 |  |
|  |  | RF | 0.82 | 0.85 | 0.85 |  |
|  |  | XGBoost | 0.82 | 0.82 | 0.83 |  |
|  | Week 9 | LR | 0.53 | 0.52 | 0.57 |  |
|  |  | GLMNET | 0.83 | 0.89 | 0.89 |  |
|  |  | RF | 0.89 | 0.87 | 0.88 |  |
|  |  | XGBoost | 0.89 | 0.88 | 0.88 |  |
| Spring 2017 | Pre | LR | 0.56 | 0.63 | 0.63 | 0.57 |
|  |  | GLMNET | 0.84 | 0.75 | 0.83 | 0.81 |
|  |  | RF | 0.62 | 0.58 | 0.73 | 0.64 |
|  |  | XGBoost | 0.58 | 0.51 | 0.52 | 0.58 |
|  | Week 3 | LR | 0.63 | 0.63 | 0.65 | 0.57 |
|  |  | GLMNET | 0.87 | 0.87 | 0.86 | 0.88 |
|  |  | RF | 0.74 | 0.78 | 0.82 | 0.79 |
|  |  | XGBoost | 0.75 | 0.81 | 0.72 | 0.83 |
|  | Week 6 | LR | 0.66 | 0.61 | 0.65 | 0.57 |
|  |  | GLMNET | 0.88 | 0.83 | 0.92 | 0.93 |
|  |  | RF | 0.84 | 0.75 | 0.81 | 0.81 |
|  |  | XGBoost | 0.84 | 0.82 | 0.73 | 0.85 |
|  | Week 9 | LR | 0.50 | 0.61 | 0.63 | 0.57 |
|  |  | GLMNET | 0.93 | 0.89 | 0.91 | 0.93 |
|  |  | RF | 0.81 | 0.84 | 0.88 | 0.89 |
|  |  | XGBoost | 0.85 | 0.88 | 0.85 | 0.84 |

Table S6: Point estimate AUC results for fall 2015 and spring 2016 testing semester across all time frames, DMMs, and corpora sizes with Correlation Attribute Evaluation

| Testing Data | Time Frame | Data Mining Method | Testing AUC: 2 semesters | Testing AUC: 3 semesters |
| --- | --- | --- | --- | --- |
| Fall 2015 | Pre | LR | 0.80 |  |
|  |  | GLMNET | 0.80 |  |
|  |  | RF | 0.72 |  |
|  |  | XGBoost | 0.72 |  |
|  | Week 3 | LR | 0.79 |  |
|  |  | GLMNET | 0.79 |  |
|  |  | RF | 0.78 |  |
|  |  | XGBoost | 0.77 |  |
|  | Week 6 | LR | 0.83 |  |
|  |  | GLMNET | 0.83 |  |
|  |  | RF | 0.80 |  |
|  |  | XGBoost | 0.79 |  |
|  | Week 9 | LR | 0.91 |  |
|  |  | GLMNET | 0.91 |  |
|  |  | RF | 0.89 |  |
|  |  | XGBoost | 0.89 |  |
| Spring 2016 | Pre | LR | 0.81 | 0.74 |
|  |  | GLMNET | 0.82 | 0.74 |
|  |  | RF | 0.72 | 0.71 |
|  |  | XGBoost | 0.68 | 0.67 |
|  | Week 3 | LR | 0.88 | 0.84 |
|  |  | GLMNET | 0.88 | 0.84 |
|  |  | RF | 0.85 | 0.71 |
|  |  | XGBoost | 0.83 | 0.71 |
|  | Week 6 | LR | 0.89 | 0.83 |
|  |  | GLMNET | 0.89 | 0.83 |
|  |  | RF | 0.87 | 0.79 |
|  |  | XGBoost | 0.85 | 0.80 |
|  | Week 9 | LR | 0.91 | 0.87 |
|  |  | GLMNET | 0.91 | 0.87 |
|  |  | RF | 0.87 | 0.83 |
|  |  | XGBoost | 0.86 | 0.85 |

Table S7: Point estimate AUC results for fall 2016 and spring 2017 testing semester across all time frames, DMMs, and corpora sizes with Correlation Attribute Evaluation

| Testing Data | Time Frame | Data Mining Method | Testing AUC: 2 semesters | Testing AUC: 3 semesters | Testing AUC: 4 semesters | Testing AUC: 5 semesters |
| --- | --- | --- | --- | --- | --- | --- |
| Fall 2016 | Pre | LR | 0.60 | 0.70 | 0.69 |  |
|  |  | GLMNET | 0.60 | 0.70 | 0.69 |  |
|  |  | RF | 0.62 | 0.66 | 0.69 |  |
|  |  | XGBoost | 0.59 | 0.62 | 0.68 |  |
|  | Week 3 | LR | 0.77 | 0.81 | 0.81 |  |
|  |  | GLMNET | 0.77 | 0.81 | 0.81 |  |
|  |  | RF | 0.76 | 0.83 | 0.76 |  |
|  |  | XGBoost | 0.75 | 0.81 | 0.73 |  |
|  | Week 6 | LR | 0.80 | 0.81 | 0.82 |  |
|  |  | GLMNET | 0.80 | 0.81 | 0.82 |  |
|  |  | RF | 0.81 | 0.82 | 0.79 |  |
|  |  | XGBoost | 0.78 | 0.79 | 0.78 |  |
|  | Week 9 | LR | 0.86 | 0.88 | 0.89 |  |
|  |  | GLMNET | 0.86 | 0.88 | 0.89 |  |
|  |  | RF | 0.86 | 0.85 | 0.88 |  |
|  |  | XGBoost | 0.86 | 0.86 | 0.86 |  |
| Spring 2017 | Pre | LR | 0.80 | 0.82 | 0.87 | 0.84 |
|  |  | GLMNET | 0.78 | 0.83 | 0.87 | 0.84 |
|  |  | RF | 0.58 | 0.63 | 0.65 | 0.66 |
|  |  | XGBoost | 0.52 | 0.63 | 0.65 | 0.63 |
|  | Week 3 | LR | 0.85 | 0.75 | 0.91 | 0.92 |
|  |  | GLMNET | 0.86 | 0.85 | 0.91 | 0.92 |
|  |  | RF | 0.70 | 0.73 | 0.76 | 0.76 |
|  |  | XGBoost | 0.56 | 0.68 | 0.77 | 0.70 |
|  | Week 6 | LR | 0.78 | 0.88 | 0.93 | 0.94 |
|  |  | GLMNET | 0.78 | 0.88 | 0.93 | 0.94 |
|  |  | RF | 0.57 | 0.71 | 0.77 | 0.83 |
|  |  | XGBoost | 0.56 | 0.70 | 0.70 | 0.80 |
|  | Week 9 | LR | 0.95 | 0.91 | 0.93 | 0.94 |
|  |  | GLMNET | 0.95 | 0.91 | 0.93 | 0.94 |
|  |  | RF | 0.93 | 0.88 | 0.85 | 0.89 |
|  |  | XGBoost | 0.87 | 0.86 | 0.86 | 0.86 |

Table S8: Point estimate AUC results for fall 2015 and spring 2016 testing semester across all time frames, DMMs, and corpora sizes with Fisher’s Scoring Algorithm

| Testing Data | Time Frame | Data Mining Method | Testing AUC: 2 semesters | Testing AUC: 3 semesters |
| --- | --- | --- | --- | --- |
| Fall 2015 | Pre | LR | 0.81 |  |
|  |  | GLMNET | 0.81 |  |
|  |  | RF | 0.73 |  |
|  |  | XGBoost | 0.71 |  |
|  | Week 3 | LR | 0.83 |  |
|  |  | GLMNET | 0.82 |  |
|  |  | RF | 0.78 |  |
|  |  | XGBoost | 0.78 |  |
|  | Week 6 | LR | 0.84 |  |
|  |  | GLMNET | 0.84 |  |
|  |  | RF | 0.80 |  |
|  |  | XGBoost | 0.79 |  |
|  | Week 9 | LR | 0.90 |  |
|  |  | GLMNET | 0.91 |  |
|  |  | RF | 0.90 |  |
|  |  | XGBoost | 0.88 |  |
| Spring 2016 | Pre | LR | 0.84 | 0.78 |
|  |  | GLMNET | 0.84 | 0.78 |
|  |  | RF | 0.76 | 0.72 |
|  |  | XGBoost | 0.71 | 0.62 |
|  | Week 3 | LR | 0.88 | 0.85 |
|  |  | GLMNET | 0.88 | 0.85 |
|  |  | RF | 0.84 | 0.77 |
|  |  | XGBoost | 0.82 | 0.79 |
|  | Week 6 | LR | 0.88 | 0.83 |
|  |  | GLMNET | 0.88 | 0.83 |
|  |  | RF | 0.86 | 0.79 |
|  |  | XGBoost | 0.86 | 0.79 |
|  | Week 9 | LR | 0.91 | 0.87 |
|  |  | GLMNET | 0.91 | 0.87 |
|  |  | RF | 0.84 | 0.83 |
|  |  | XGBoost | 0.85 | 0.85 |

Table S9: Point estimate AUC results for fall 2016 and spring 2017 testing semester across all time frames, DMMs, and corpora sizes with Fisher’s Scoring Algorithm

| Testing Data | Time Frame | Data Mining Method | Testing AUC: 2 semesters | Testing AUC: 3 semesters | Testing AUC: 4 semesters | Testing AUC: 5 semesters |
| --- | --- | --- | --- | --- | --- | --- |
| Fall 2016 | Pre | LR | 0.69 | 0.71 | 0.73 |  |
|  |  | GLMNET | 0.68 | 0.71 | 0.73 |  |
|  |  | RF | 0.73 | 0.65 | 0.71 |  |
|  |  | XGBoost | 0.72 | 0.62 | 0.70 |  |
|  | Week 3 | LR | 0.83 | 0.83 | 0.83 |  |
|  |  | GLMNET | 0.83 | 0.82 | 0.83 |  |
|  |  | RF | 0.80 | 0.83 | 0.77 |  |
|  |  | XGBoost | 0.79 | 0.81 | 0.69 |  |
|  | Week 6 | LR | 0.79 | 0.84 | 0.93 |  |
|  |  | GLMNET | 0.78 | 0.84 | 0.93 |  |
|  |  | RF | 0.78 | 0.82 | 0.76 |  |
|  |  | XGBoost | 0.75 | 0.80 | 0.67 |  |
|  | Week 9 | LR | 0.87 | 0.89 | 0.94 |  |
|  |  | GLMNET | 0.87 | 0.89 | 0.93 |  |
|  |  | RF | 0.88 | 0.88 | 0.87 |  |
|  |  | XGBoost | 0.88 | 0.87 | 0.85 |  |
| Spring 2017 | Pre | LR | 0.84 | 0.84 | 0.87 | 0.84 |
|  |  | GLMNET | 0.84 | 0.84 | 0.87 | 0.85 |
|  |  | RF | 0.68 | 0.63 | 0.64 | 0.69 |
|  |  | XGBoost | 0.60 | 0.63 | 0.64 | 0.64 |
|  | Week 3 | LR | 0.83 | 0.90 | 0.92 | 0.92 |
|  |  | GLMNET | 0.83 | 0.90 | 0.92 | 0.92 |
|  |  | RF | 0.71 | 0.78 | 0.76 | 0.78 |
|  |  | XGBoost | 0.65 | 0.78 | 0.78 | 0.75 |
|  | Week 6 | LR | 0.87 | 0.91 | 0.93 | 0.94 |
|  |  | GLMNET | 0.86 | 0.91 | 0.93 | 0.94 |
|  |  | RF | 0.80 | 0.77 | 0.76 | 0.81 |
|  |  | XGBoost | 0.77 | 0.77 | 0.67 | 0.81 |
|  | Week 9 | LR | 0.95 | 0.91 | 0.94 | 0.95 |
|  |  | GLMNET | 0.95 | 0.91 | 0.93 | 0.95 |
|  |  | RF | 0.93 | 0.86 | 0.87 | 0.89 |
|  |  | XGBoost | 0.87 | 0.87 | 0.85 | 0.84 |

Table S10: Point estimate AUC results for fall 2015 and spring 2016 testing semester across all time frames, DMMs, and corpora sizes with Information Gain Attribute Evaluation

| Testing Data | Time Frame | Data Mining Method | Testing AUC: 2 semesters | Testing AUC: 3 semesters |
| --- | --- | --- | --- | --- |
| Fall 2015 | Pre | LR | 0.69 |  |
|  |  | GLMNET | 0.69 |  |
|  |  | RF | 0.69 |  |
|  |  | XGBoost | 0.70 |  |
|  | Week 3 | LR | 0.64 |  |
|  |  | GLMNET | 0.76 |  |
|  |  | RF | 0.69 |  |
|  |  | XGBoost | 0.68 |  |
|  | Week 6 | LR | 0.71 |  |
|  |  | GLMNET | 0.71 |  |
|  |  | RF | 0.71 |  |
|  |  | XGBoost | 0.71 |  |
|  | Week 9 | LR | 0.90 |  |
|  |  | GLMNET | 0.90 |  |
|  |  | RF | 0.87 |  |
|  |  | XGBoost | 0.85 |  |
| Spring 2016 | Pre | LR | 0.75 | 0.76 |
|  |  | GLMNET | 0.75 | 0.76 |
|  |  | RF | 0.66 | 0.61 |
|  |  | XGBoost | 0.60 | 0.54 |
|  | Week 3 | LR | 0.76 | 0.76 |
|  |  | GLMNET | 0.84 | 0.84 |
|  |  | RF | 0.76 | 0.61 |
|  |  | XGBoost | 0.61 | 0.62 |
|  | Week 6 | LR | 0.84 | 0.73 |
|  |  | GLMNET | 0.84 | 0.87 |
|  |  | RF | 0.79 | 0.70 |
|  |  | XGBoost | 0.79 | 0.67 |
|  | Week 9 | LR | 0.91 | 0.85 |
|  |  | GLMNET | 0.91 | 0.85 |
|  |  | RF | 0.85 | 0.79 |
|  |  | XGBoost | 0.82 | 0.81 |

Table S11: Point estimate AUC results for fall 2016 and spring 2017 testing semester across all time frames, DMMs, and corpora sizes with Information Gain Attribute Evaluation

| Testing Data | Time Frame | Data Mining Method | Testing AUC: 2 semesters | Testing AUC: 3 semesters | Testing AUC: 4 semesters | Testing AUC:  5 semesters |
| --- | --- | --- | --- | --- | --- | --- |
| Fall 2016 | Pre | LR | 0.69 | 0.65 | 0.73 |  |
|  |  | GLMNET | 0.69 | 0.65 | 0.73 |  |
|  |  | RF | 0.69 | 0.65 | 0.70 |  |
|  |  | XGBoost | 0.66 | 0.62 | 0.67 |  |
|  | Week 3 | LR | 0.74 | 0.61 | 0.78 |  |
|  |  | GLMNET | 0.74 | 0.80 | 0.78 |  |
|  |  | RF | 0.73 | 0.70 | 0.65 |  |
|  |  | XGBoost | 0.70 | 0.64 | 0.67 |  |
|  | Week 6 | LR | 0.79 | 0.68 | 0.81 |  |
|  |  | GLMNET | 0.79 | 0.83 | 0.81 |  |
|  |  | RF | 0.73 | 0.76 | 0.77 |  |
|  |  | XGBoost | 0.73 | 0.76 | 0.81 |  |
|  | Week 9 | LR | 0.82 | 0.64 | 0.89 |  |
|  |  | GLMNET | 0.82 | 0.87 | 0.89 |  |
|  |  | RF | 0.76 | 0.79 | 0.82 |  |
|  |  | XGBoost | 0.74 | 0.81 | 0.82 |  |
| Spring 2017 | Pre | LR | 0.66 | 0.77 | 0.65 | 0.83 |
|  |  | GLMNET | 0.62 | 0.77 | 0.65 | 0.84 |
|  |  | RF | 0.51 | 0.58 | 0.56 | 0.63 |
|  |  | XGBoost | 0.56 | 0.60 | 0.60 | 0.56 |
|  | Week 3 | LR | 0.79 | 0.85 | 0.81 | 0.89 |
|  |  | GLMNET | 0.81 | 0.84 | 0.81 | 0.89 |
|  |  | RF | 0.69 | 0.71 | 0.57 | 0.72 |
|  |  | XGBoost | 0.69 | 0.77 | 0.60 | 0.75 |
|  | Week 6 | LR | 0.89 | 0.85 | 0.81 | 0.91 |
|  |  | GLMNET | 0.89 | 0.85 | 0.87 | 0.91 |
|  |  | RF | 0.78 | 0.71 | 0.87 | 0.68 |
|  |  | XGBoost | 0.80 | 0.74 | 0.52 | 0.59 |
|  | Week 9 | LR | 0.86 | 0.87 | 0.91 | 0.94 |
|  |  | GLMNET | 0.87 | 0.87 | 0.91 | 0.94 |
|  |  | RF | 0.68 | 0.63 | 0.74 | 0.83 |
|  |  | XGBoost | 0.71 | 0.64 | 0.78 | 0.84 |

Table S12: Point estimate AUC results for fall 2015 and spring 2016 testing semester across all time frames, DMMs, and corpora sizes with Relief Attribute Evaluation

| Testing Data | Time Frame | Data Mining Method | Testing AUC: 2 semesters | Testing AUC: 3 semesters |
| --- | --- | --- | --- | --- |
| Fall 2015 | Pre | LR | 0.77 |  |
|  |  | GLMNET | 0.77 |  |
|  |  | RF | 0.70 |  |
|  |  | XGBoost | 0.65 |  |
|  | Week 3 | LR | 0.75 |  |
|  |  | GLMNET | 0.75 |  |
|  |  | RF | 0.68 |  |
|  |  | XGBoost | 0.65 |  |
|  | Week 6 | LR | 0.68 |  |
|  |  | GLMNET | 0.69 |  |
|  |  | RF | 0.62 |  |
|  |  | XGBoost | 0.60 |  |
|  | Week 9 | LR | 0.68 |  |
|  |  | GLMNET | 0.68 |  |
|  |  | RF | 0.65 |  |
|  |  | XGBoost | 0.60 |  |
| Spring 2016 | Pre | LR | 0.73 | 0.79 |
|  |  | GLMNET | 0.73 | 0.80 |
|  |  | RF | 0.72 | 0.68 |
|  |  | XGBoost | 0.73 | 0.56 |
|  | Week 3 | LR | 0.80 | 0.64 |
|  |  | GLMNET | 0.80 | 0.64 |
|  |  | RF | 0.62 | 0.60 |
|  |  | XGBoost | 0.55 | 0.61 |
|  | Week 6 | LR | 0.86 | 0.73 |
|  |  | GLMNET | 0.86 | 0.72 |
|  |  | RF | 0.84 | 0.60 |
|  |  | XGBoost | 0.79 | 0.53 |
|  | Week 9 | LR | 0.92 | 0.72 |
|  |  | GLMNET | 0.92 | 0.72 |
|  |  | RF | 0.90 | 0.70 |
|  |  | XGBoost | 0.86 | 0.55 |

Table S13: Point estimate AUC results for fall 2015 and spring 2016 testing semester across all time frames, DMMs, and corpora sizes with Relief Attribute Evaluation

| Testing Data | Time Frame | Data Mining Method | Testing AUC: 2 semesters | Testing AUC: 3 semesters | Testing AUC: 4 semesters | Testing AUC: 5 semesters |
| --- | --- | --- | --- | --- | --- | --- |
| Fall 2016 | Pre | LR | 0.65 | 0.69 | 0.62 |  |
|  |  | GLMNET | 0.64 | 0.69 | 0.62 |  |
|  |  | RF | 0.65 | 0.69 | 0.64 |  |
|  |  | XGBoost | 0.67 | 0.70 | 0.61 |  |
|  | Week 3 | LR | 0.72 | 0.74 | 0.75 |  |
|  |  | GLMNET | 0.72 | 0.75 | 0.75 |  |
|  |  | RF | 0.72 | 0.69 | 0.66 |  |
|  |  | XGBoost | 0.72 | 0.69 | 0.62 |  |
|  | Week 6 | LR | 0.62 | 0.72 | 0.71 |  |
|  |  | GLMNET | 0.62 | 0.72 | 0.71 |  |
|  |  | RF | 0.63 | 0.71 | 0.68 |  |
|  |  | XGBoost | 0.60 | 0.69 | 0.66 |  |
|  | Week 9 | LR | 0.81 | 0.83 | 0.80 |  |
|  |  | GLMNET | 0.82 | 0.83 | 0.80 |  |
|  |  | RF | 0.75 | 0.81 | 0.50 |  |
|  |  | XGBoost | 0.73 | 0.77 | 0.51 |  |
| Spring 2017 | Pre | LR | 0.68 | 0.59 | 0.83 | 0.61 |
|  |  | GLMNET | 0.66 | 0.60 | 0.82 | 0.61 |
|  |  | RF | 0.61 | 0.56 | 0.57 | 0.64 |
|  |  | XGBoost | 0.62 | 0.69 | 0.56 | 0.61 |
|  | Week 3 | LR | 0.77 | 0.72 | 0.85 | 0.61 |
|  |  | GLMNET | 0.77 | 0.72 | 0.86 | 0.62 |
|  |  | RF | 0.72 | 0.68 | 0.51 | 0.52 |
|  |  | XGBoost | 0.53 | 0.58 | 0.54 | 0.53 |
|  | Week 6 | LR | 0.62 | 0.60 | 0.88 | 0.76 |
|  |  | GLMNET | 0.61 | 0.59 | 0.88 | 0.76 |
|  |  | RF | 0.58 | 0.56 | 0.85 | 0.57 |
|  |  | XGBoost | 0.75 | 0.54 | 0.83 | 0.61 |
|  | Week 9 | LR | 0.87 | 0.62 | 0.69 | 0.88 |
|  |  | GLMNET | 0.87 | 0.62 | 0.69 | 0.88 |
|  |  | RF | 0.73 | 0.63 | 0.57 | 0.78 |
|  |  | XGBoost | 0.61 | 0.53 | 0.53 | 0.75 |

Table S14: SC metric for fall 2015 and spring 2016 testing corpus

| Testing Data Corpus | Time Frame | Pre-Processing Feature Selection Technique | Training Data: 2 semesters | Training Data: 3 semesters |
| --- | --- | --- | --- | --- |
| Fall 2015 | Pre | CAE | 0.86 |  |
|  |  | FSA | 0.93 |  |
|  |  | IG | 0.91 |  |
|  |  | RAE | 0.23 |  |
|  | Week 3 | CAE | 0.94 |  |
|  |  | FSA | 1.00 |  |
|  |  | IG | 0.85 |  |
|  |  | RAE | 0.31 |  |
|  | Week 6 | CAE | 1.00 |  |
|  |  | FSA | 1.00 |  |
|  |  | IG | 0.73 |  |
|  |  | RAE | 0.20 |  |
|  | Week 9 | CAE | 1.00 |  |
|  |  | FSA | 0.90 |  |
|  |  | IG | 0.87 |  |
|  |  | RAE | 0.18 |  |
| Spring 2016 | Pre | CAE | 0.91 | 1.00 |
|  |  | FSA | 0.91 | 1.00 |
|  |  | IG | 0.90 | 0.90 |
|  |  | RAE | 0.25 | 0.19 |
|  | Week 3 | CAE | 0.90 | 0.90 |
|  |  | FSA | 0.96 | 0.90 |
|  |  | IG | 0.79 | 0.85 |
|  |  | RAE | 0.09 | 0.18 |
|  | Week 6 | CAE | 1.00 | 1.00 |
|  |  | FSA | 0.84 | 0.87 |
|  |  | IG | 0.85 | 0.81 |
|  |  | RAE | 0.20 | 0.20 |
|  | Week 9 | CAE | 1.00 | 1.00 |
|  |  | FSA | 1.00 | 1.00 |
|  |  | IG | 0.90 | 0.88 |
|  |  | RAE | 0.15 | 0.10 |

Table S15: SC metric for fall 2016 and spring 2017 testing corpus

| Testing Data Corpus | Time Frame | Pre-Processing Feature Selection Technique | Training Data: 2 semesters | Training Data: 3 semesters | Training Data: 4 semesters | Training Data: 5 semesters |
| --- | --- | --- | --- | --- | --- | --- |
| Fall 2016 | Pre | CAE | 1.00 | 1.00 | 0.96 |  |
|  |  | FSA | 0.90 | 1.00 | 0.87 |  |
|  |  | IG | 0.81 | 0.85 | 0.93 |  |
|  |  | RAE | 0.15 | 0.23 | 0.24 |  |
|  | Week 3 | CAE | 0.91 | 1.00 | 0.91 |  |
|  |  | FSA | 0.94 | 1.00 | 1.00 |  |
|  |  | IG | 0.73 | 0.86 | 0.88 |  |
|  |  | RAE | 0.15 | 0.13 | 0.18 |  |
|  | Week 6 | CAE | 1.00 | 1.00 | 1.00 |  |
|  |  | FSA | 0.94 | 0.93 | 0.89 |  |
|  |  | IG | 0.73 | 0.76 | 0.85 |  |
|  |  | RAE | 0.12 | 0.14 | 0.19 |  |
|  | Week 9 | CAE | 0.86 | 1.00 | 1.00 |  |
|  |  | FSA | 1.00 | 1.00 | 1.00 |  |
|  |  | IG | 0.78 | 0.96 | 0.88 |  |
|  |  | RAE | 0.13 | 0.31 | 0.25 |  |
| Spring 2017 | Pre | CAE | 0.96 | 0.91 | 1.00 | 0.96 |
|  |  | FSA | 0.96 | 0.90 | 1.00 | 1.00 |
|  |  | IG | 0.82 | 0.93 | 0.86 | 0.81 |
|  |  | RAE | 0.34 | 0.19 | 0.25 | 0.16 |
|  | Week 3 | CAE | 1.00 | 0.90 | 0.87 | 0.89 |
|  |  | FSA | 0.96 | 1.00 | 1.00 | 0.90 |
|  |  | IG | 0.85 | 0.93 | 0.88 | 0.74 |
|  |  | RAE | 0.24 | 0.15 | 0.17 | 0.13 |
|  | Week 6 | CAE | 0.89 | 0.94 | 0.96 | 0.90 |
|  |  | FSA | 1.00 | 1.00 | 1.00 | 0.94 |
|  |  | IG | 0.96 | 0.88 | 0.85 | 0.79 |
|  |  | RAE | 0.20 | 0.18 | 0.21 | 0.16 |
|  | Week 9 | CAE | 1.00 | 0.91 | 0.90 | 0.89 |
|  |  | FSA | 0.91 | 1.00 | 1.00 | 1.00 |
|  |  | IG | 0.96 | 0.87 | 0.80 | 0.81 |
|  |  | RAE | 0.23 | 0.23 | 0.15 | 0.24 |

**C: Top six ranked predictors selected by each preprocessing technique across all training corpora**

Figure S1: Top six ranked predictors: two training semesters and fall 2015 testing semester – pre-course


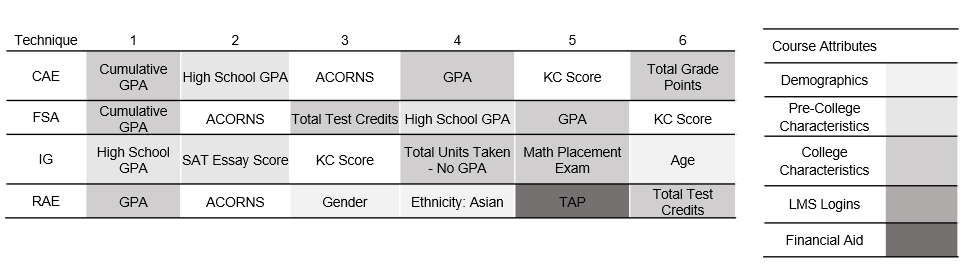


Figure S2: Top six ranked predictors: two training semesters and fall 2015 testing semester – week 3


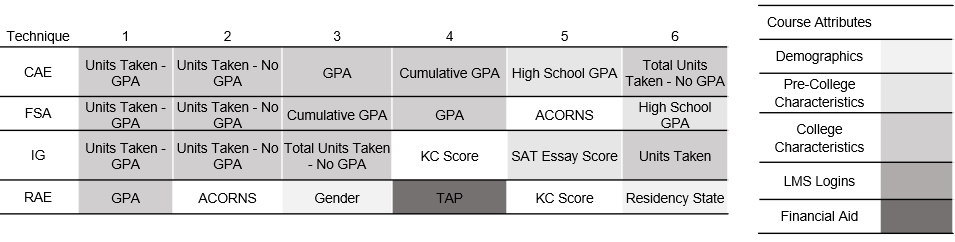


Figure S3: Top six ranked predictors: two training semesters and fall 2015 testing semester – week 6
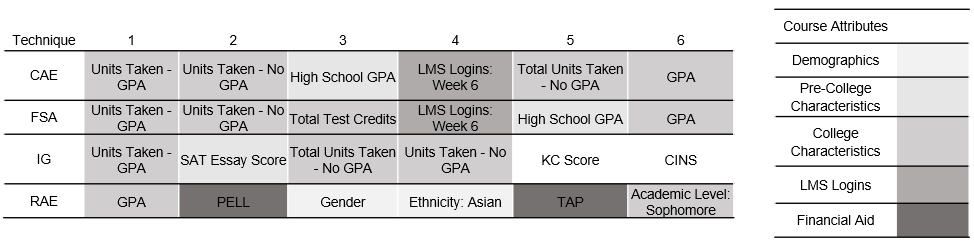


Figure S4: Top six ranked predictors: two training semesters and fall 2015 testing semester – week 9


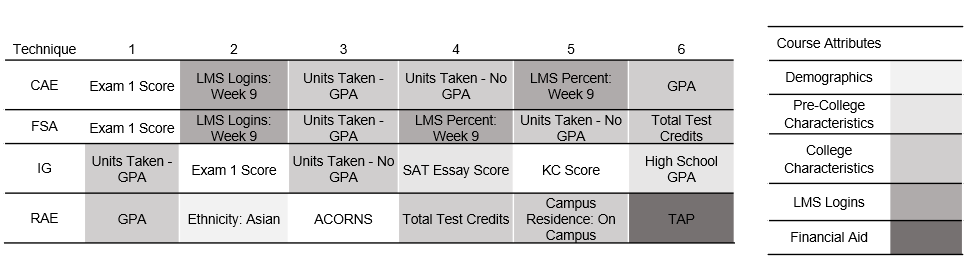


Figure S5: Top six ranked predictors: two training semesters and spring 2016 testing semester – pre-course


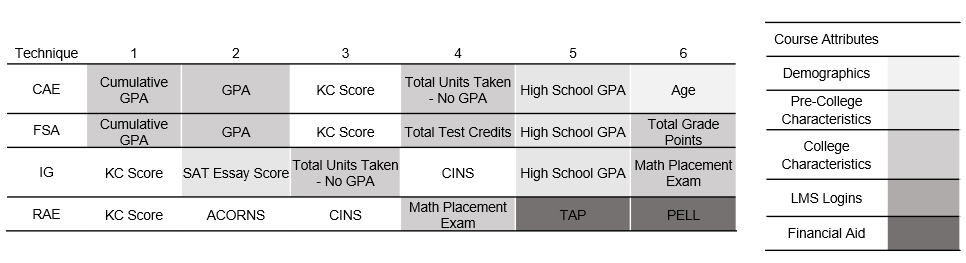


Figure S6: Top six ranked predictors: two training semesters and spring 2016 testing semester – week 3
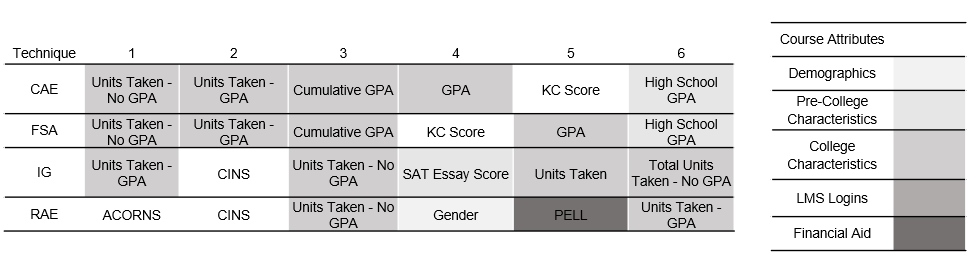


Figure S7: Top six ranked predictors: two training semesters and spring 2016 testing semester – week 6


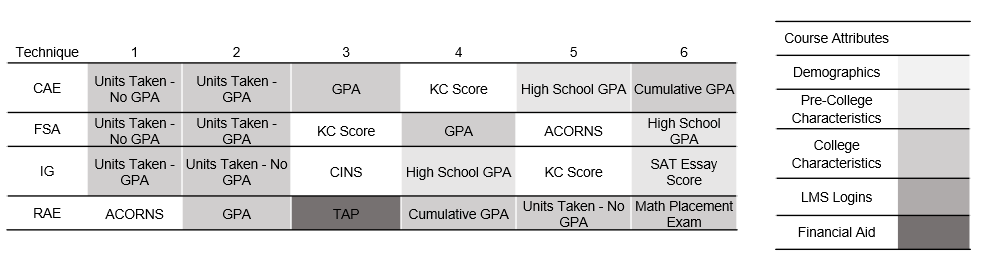


Figure S8: Top six ranked predictors: two training semesters and spring 2016 testing semester – week 9
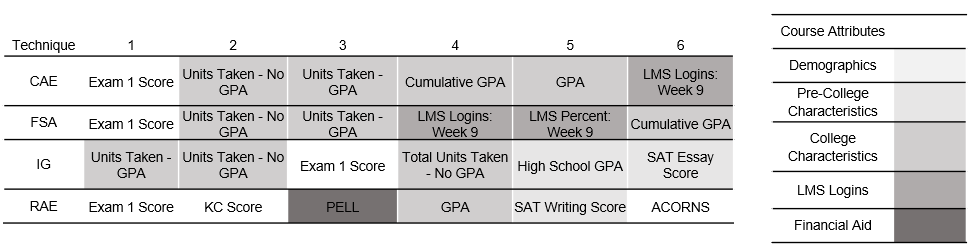


Figure S9: Top six ranked predictors: two training semesters and fall 2016 testing semester – pre-course


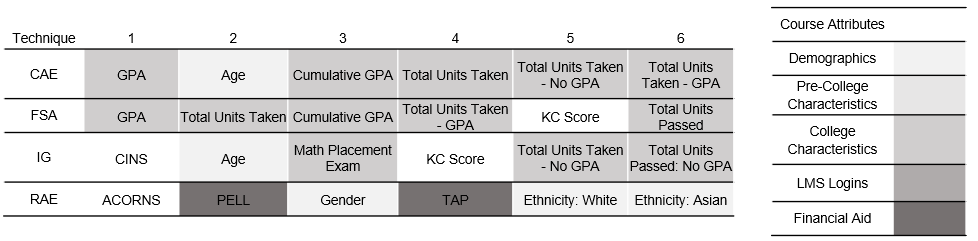


Figure S10: Top six ranked predictors: two training semesters and fall 2016 testing semester – week 3


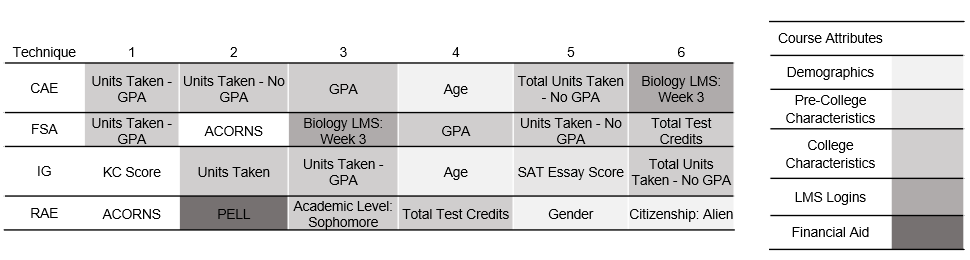


Figure S11: Top six ranked predictors: two training semesters and fall 2016 testing semester – week 6


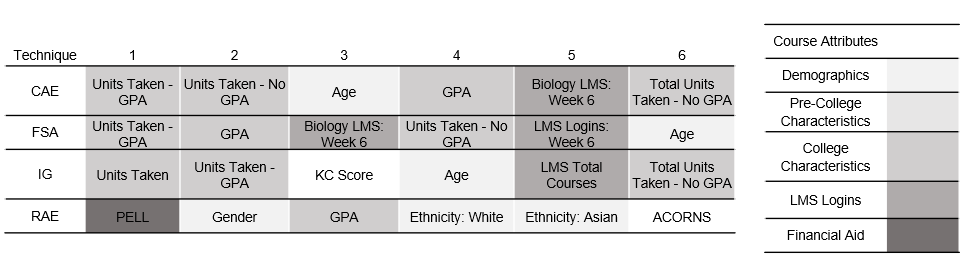


Figure S12: Top six ranked predictors: two training semesters and fall 2016 testing semester – week 9


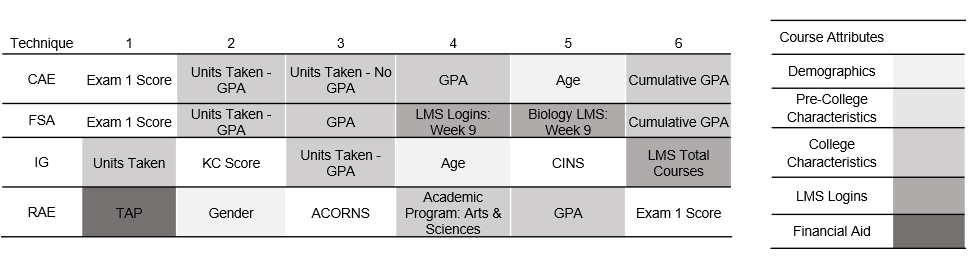


Figure S13: Top six ranked predictors: two training semesters and spring 2017 testing semester – pre-course


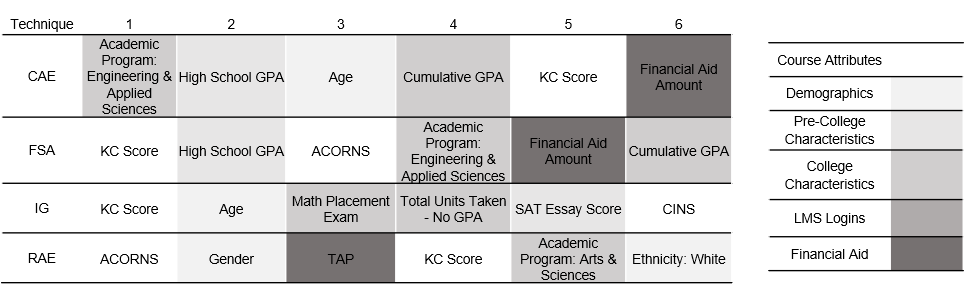


Figure S14: Top six ranked predictors: two training semesters and spring 2017 testing semester – week 3


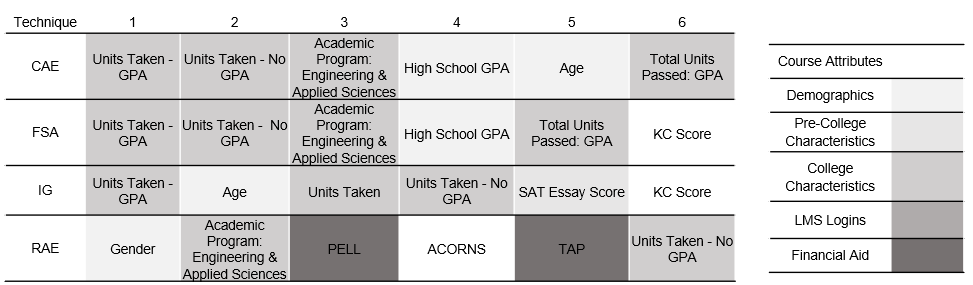


Figure S15: Top six ranked predictors: two training semesters and spring 2017 testing semester – week 6


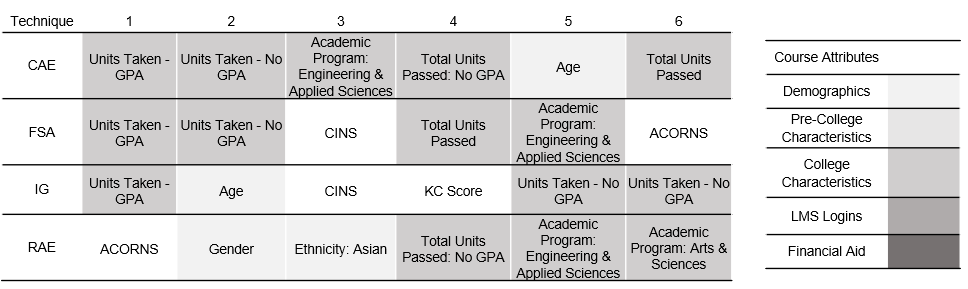


Figure S16: Top six ranked predictors: two training semesters and spring 2017 testing semester – week 9


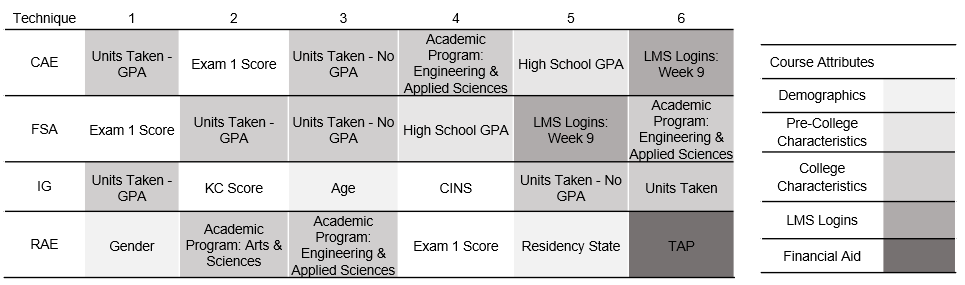


Figure S17: Top six ranked predictors: three training semesters and spring 2016 testing semester – pre-course


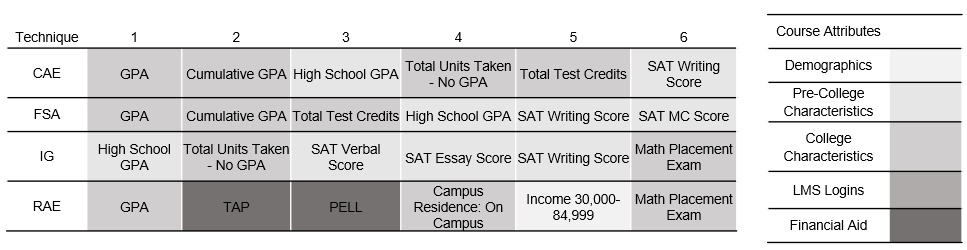


Figure S18: Top six ranked predictors: three training semesters and spring 2016 testing semester – week 3


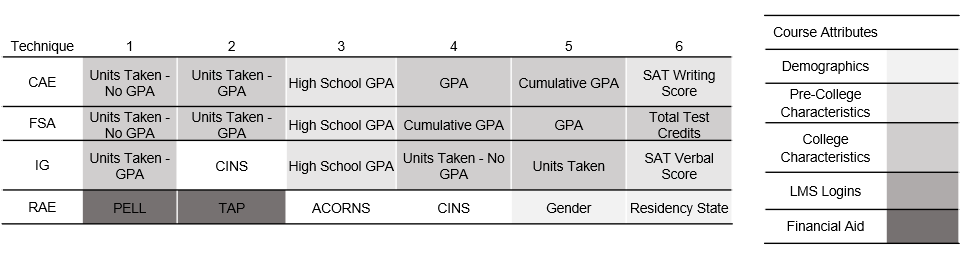


Figure S19: Top six ranked predictors: three training semesters and spring 2016 testing semester – week 6


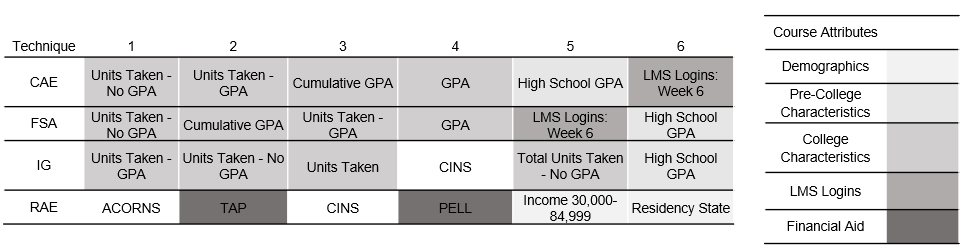


Figure S20: Top six ranked predictors: three training semesters and spring 2016 testing semester – week 9


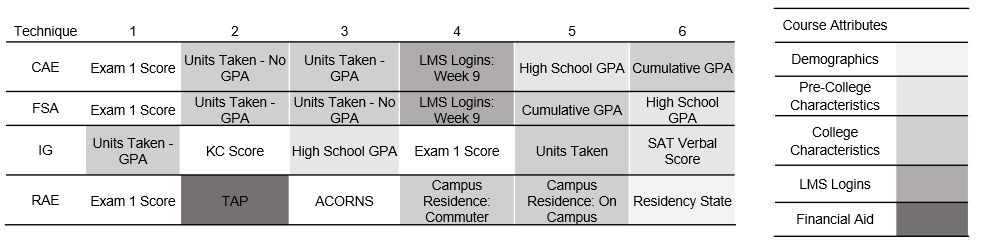


Figure S21: Top six ranked predictors: three training semesters and fall 2016 testing semester - pre-course


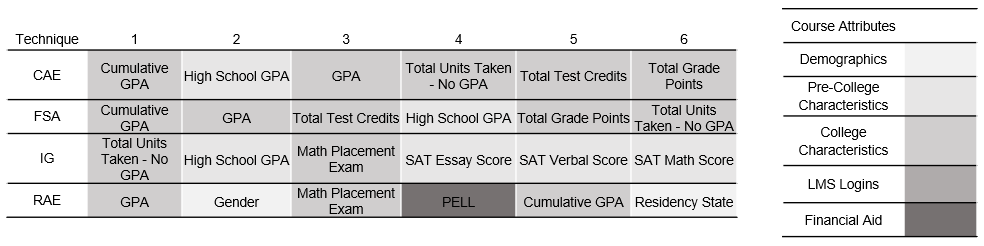


Figure S22: Top six ranked predictors: three training semesters and fall 2016 testing semester – week 3


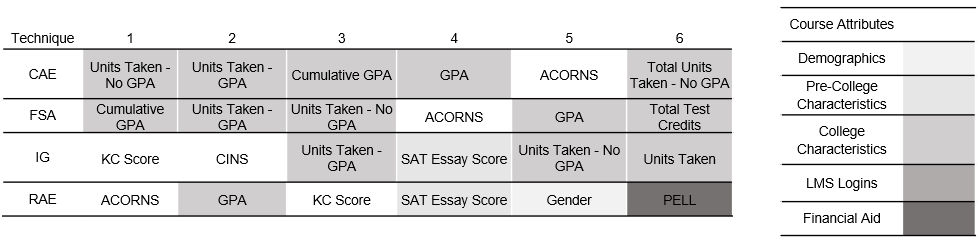


Figure S23: Top six ranked predictors: three training semesters and fall 2016 testing semester – week 6


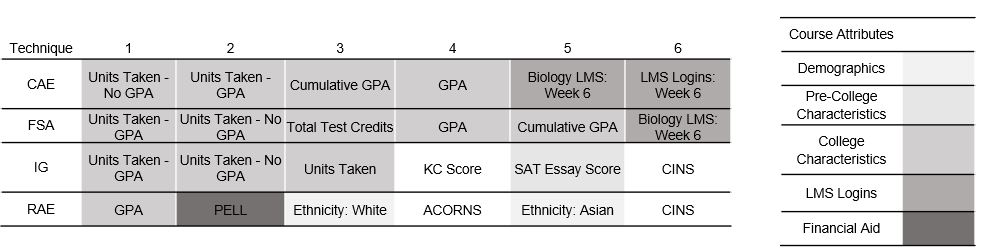


Figure S24: Top six ranked predictors: three training semesters and fall 2016 testing semester – week 9


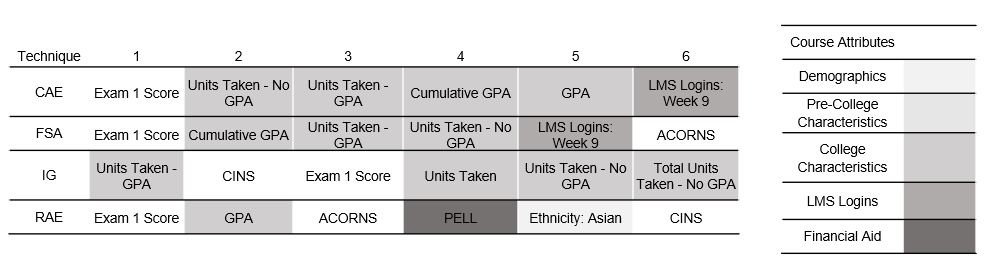


Figure S25: Top six ranked predictors: three training semesters and spring 2017 testing semester - pre-course


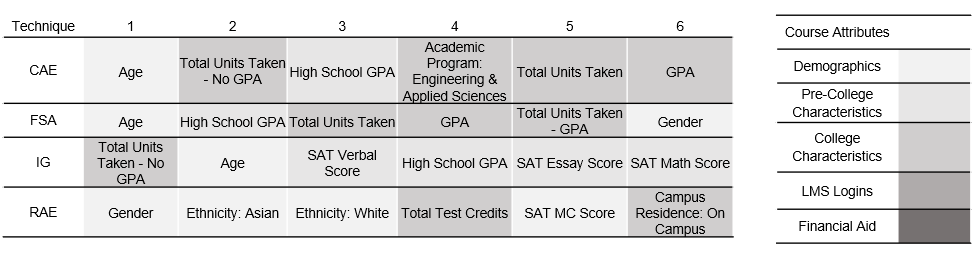


Figure S26: Top six ranked predictors: three training semesters and spring 2017 testing semester – week 3


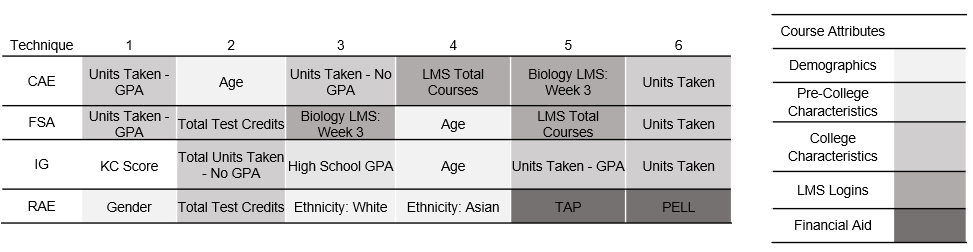


Figure S27: Top six ranked predictors: three training semesters and spring 2017 testing semester – week 6


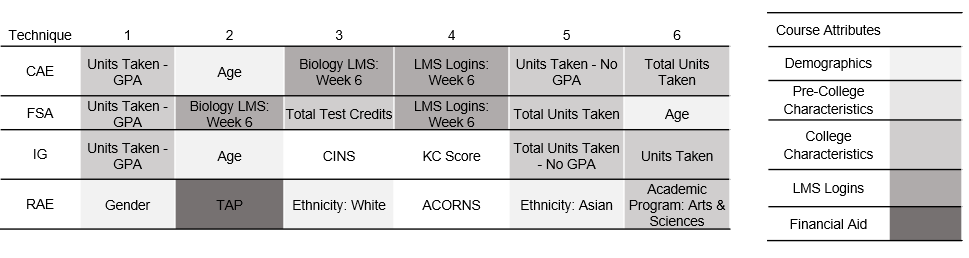


Figure S28: Top six ranked predictors: three training semesters and spring 2017 testing semester – week 9


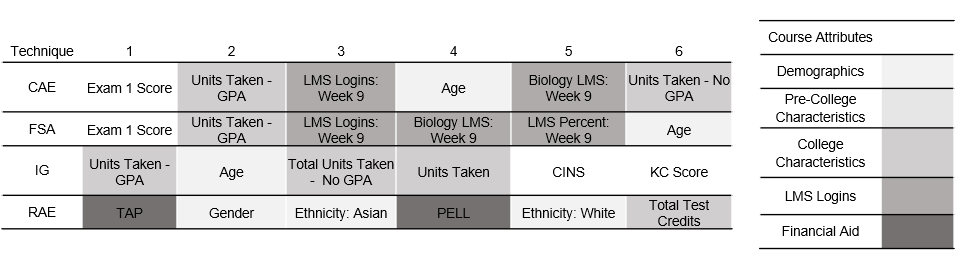


Figure S29: Top six ranked predictors: four training semesters and fall 2016 testing semester – pre-course


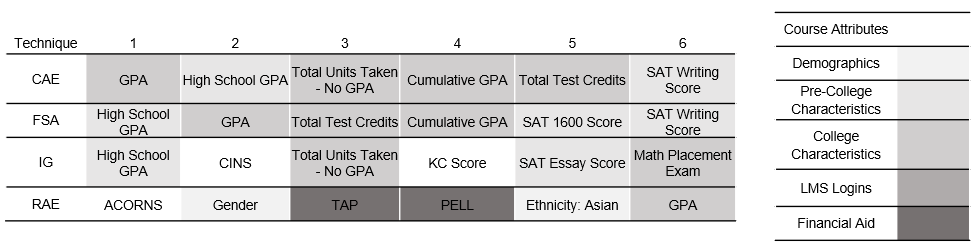


Figure S30: Top six ranked predictors: four training semesters and fall 2016 testing semester – week 3


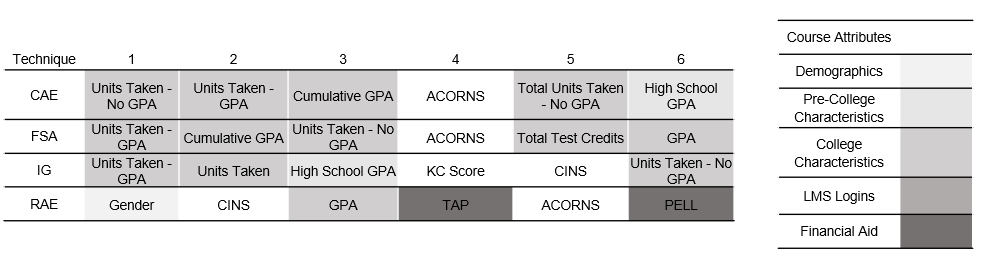


Figure S31: Top six ranked predictors: four training semesters and fall 2016 testing semester – week 6


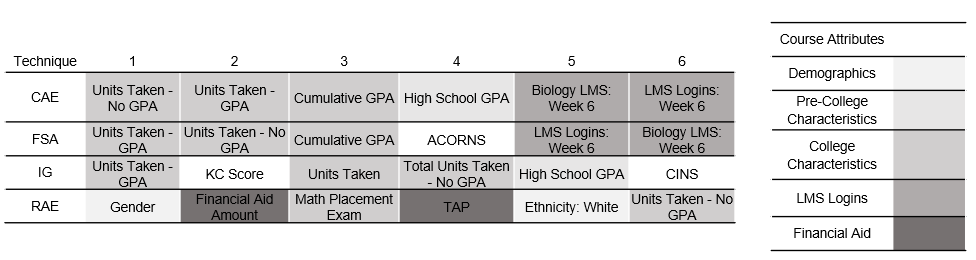


Figure S32: Top six ranked predictors: four training semesters and fall 2016 testing semester – week 9


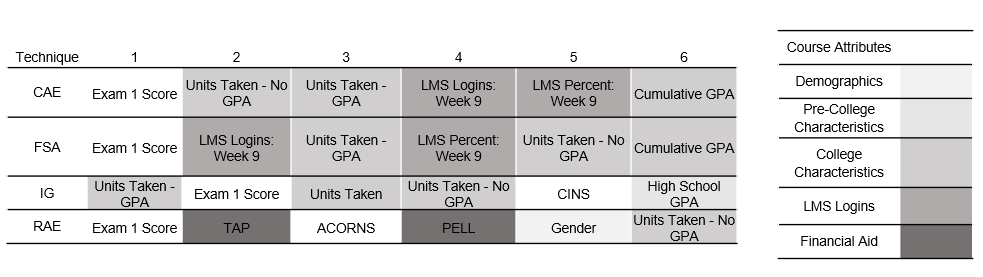


Figure S33: Top six ranked predictors: four training semesters and spring 2017 testing semester – pre-course


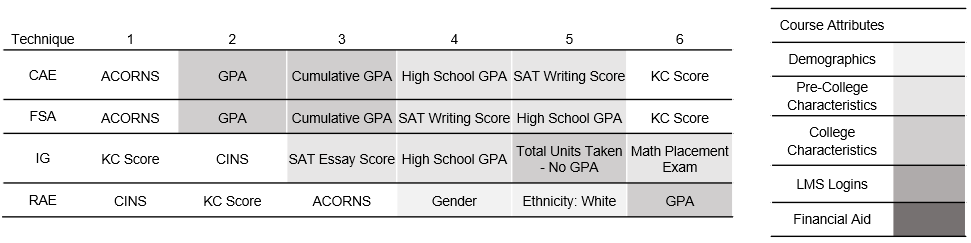


Figure S34: Top six ranked predictors: four training semesters and spring 2017 testing semester – week 3


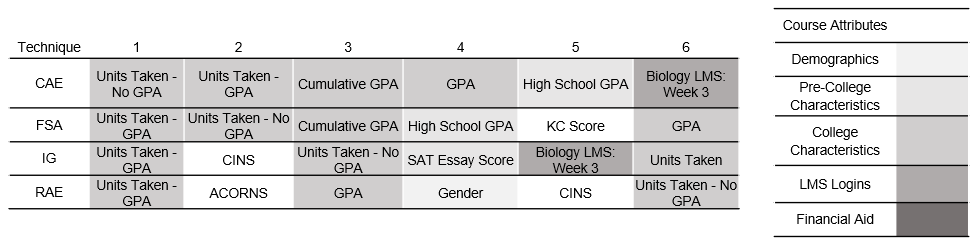


Figure S35: Top six ranked predictors: four training semesters and spring 2017 testing semester – week 6


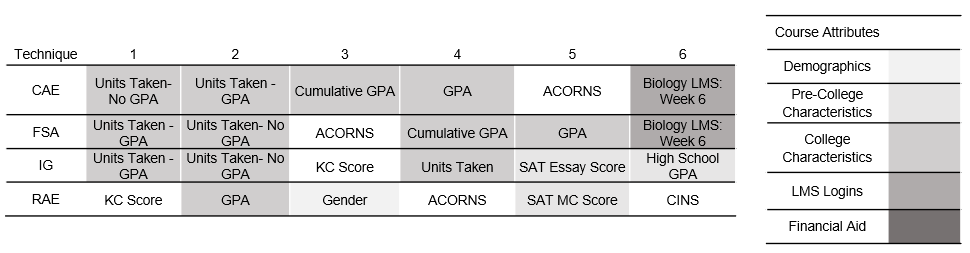


Figure S36: Top six ranked predictors: four training semesters and spring 2017 testing semester – week 9


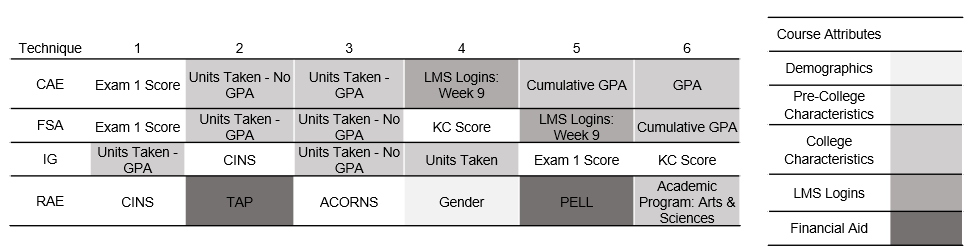


Figure S37: Top six ranked predictors: five training semesters and spring 2017 testing semester – pre-course


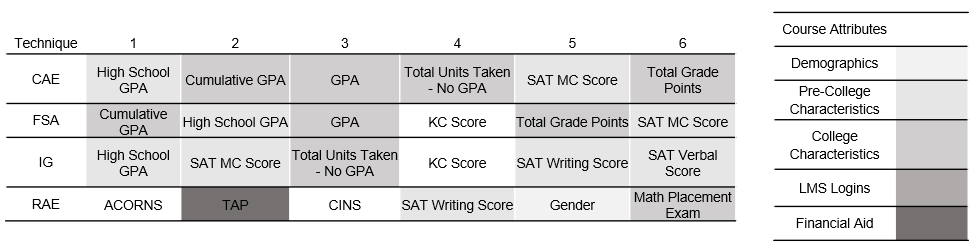


Figure S38: Top six ranked predictors: five training semesters and spring 2017 testing semester – week 3


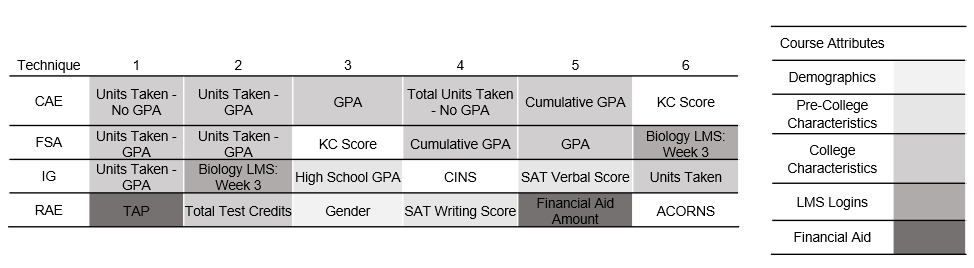


Figure S39: Top six ranked predictors: five training semesters and spring 2017 testing semester – week 6


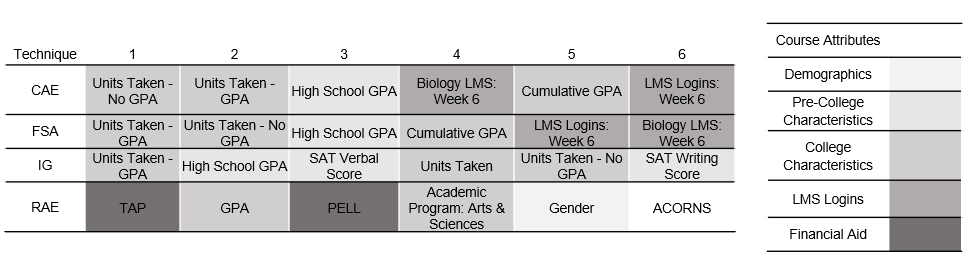


Figure S40: Top six ranked predictors: five training semesters and spring 2017 testing semester – week 9


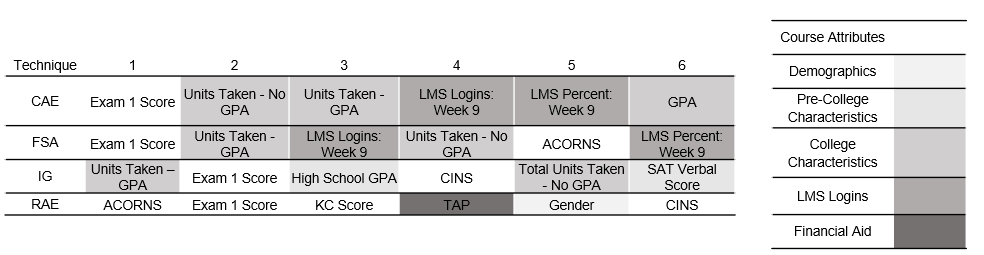


**D: Description of Preprocessing Feature Selection Techniques**

**Correlation Attribute Evaluation (CAE)**

This method calculates the Pearson correlation coefficient between each independent variable and the dependent variable and ranks features in order of their absolute correlation coefficient (Holmes et al., 1994; Hall, 1999):

Equation 1: Correlation Attribute Evaluation Formula

$$p_{i}=\left| \frac{cov\left( X_{i},Y \right)}{\sigma_{X_{i}}\sigma_{Y}} \right|$$

**Fisher’s Scoring Algorithm (FSA)**

For the case of a binary dependent variable with positive class $j=1$and negative class $j=0$and $i=1,\ldots,m$ predictors, features are ranked by the metric:

Equation 2: Fisher’s Scoring Algorithm for Binary Classification

$$S_{i}= \frac{n_{1}{(\mu_{i,1}-\mu_{i})}^{2}+n_{0}{(\mu_{i,0}-\mu_{i})}^{2}}{\left( n_{1}-1 \right)\sigma_{i,1}^{2}+(n_{0}-1)\sigma_{i,0}^{2}}$$

$n_{j}$ denotes the number of observations in the *j*^th^ class. $\mu_{ij}$ and $\sigma_{ij}^{2}$ are the mean and variance of the *i*^th^ variable in the *j*^th^ class, respectively, and $\mu_{i}$ is the overall mean of the *i*^th^ feature. However, this technique is unable to detect multicollinearity between predictors (Duda et al., 2003; Tang et al., 2014).

**Information Gain Attribute Evaluation (IG)**

This algorithm identifies predictors highly correlated with the dependent variable but unlike CAE, selects independent variables that have low correlations among themselves (Mangal, 2018). The drawback to this technique is that it tends to select attributes with a high variance (Vege, 2012).

IG quantifies the decrease in entropy of the independent variable based on the dependent variable:

Equation 3: Information Gain Formula

$$IG\left( X,Y \right)=H\left( X \right)-H(X|Y)$$

where entropy is a measurement of uncertainty and $p(x_{i})$ is the prior probability for all values of the random variable $X$.

Condition entropy is defined as:

Equation 4: Conditional Entropy Formula

$$H\left( X | Y \right)= -\sum_{i} p\left( y_{i} \right)\sum_{j} p\left( x_{i} | y_{j} \right)\log_{2} p\left( x_{i} | y_{j} \right)$$

An IG of 0 corresponds to independence between $X$ and $Y$, while an IG of 1 means that one variable can be used to completely predict the other. This method requires the data to be normalized to ensure prediction rankings are comparable.

**Relief Attribute Evaluation (RAE)**

This method is the only individual filter feature selection algorithm capable of detecting interactions between predictors via a nearest neighbor technique (Bolón-Canedo et al., 2013). This method does not explicitly tabulate an optimal subset of predictors but estimates the merit of each feature in the context of the other features and observations in each class (Kira and Rendell, 1992).

The algorithm calculates a feature weight *W* between -1 (worst) and 1(best) to assess the relevance of each covariate to the target outcome. A set of *k* random training points are selected, without replacement, denoted by $R_{i}$and the weight *W* is updated based on the feature value difference between the *k* sampled points and two neighboring instances: the nearest hit (*H*) observation, belonging to the same class, and nearest miss (*M*) observation, belonging to the opposing class. The weight of the feature increases by $\frac{1}{k}$ for instance *M* and reduces by $\frac{1}{k}$ for instance *H* (Robnik-Šikonja and Kononenko, 2001; Urbanowicz et al., 2018). For categorical features, this difference is used to tabulate the distance between observations for feature *A* between $R_{i}$ and *I* (where *I* is either *H* or *M*) defined by:

Equation 5: Relief Distance Function for Categorical Features

$$diff\left( A,R_{i},I \right)= \left\{ \begin{aligned} 1, otherwise \\ 0, value\left( A{,R}_{i} \right)=value(A,I) \end{aligned} \right.$$

The difference function for continuous predictors is defined by:

Equation 6: Relief Distance Function for Continuous Features

$$diff\left( A,R_{i},I \right)= \frac{|value\left( A{,R}_{i} \right)-value(A,I)|}{\max\left( A \right)-min(A)}$$

**E: Concept inventory assessments**

Three of the four biology course features came from two concept inventory (CI) assessments administered before the start of the course to assess a student’s prior knowledge of evolutionary theory: the ACORNS (Assessing Contextual Reasoning about Natural Selection, Nehm et al., 2012) and the CINS (Conceptual Inventory of Natural Selection, Anderson et al., 2002). CI tools are used by instructors to address conceptual difficulties (e.g., misconceptions of course content and prerequisite concepts) and diagnose student levels of scientific understanding (Haudek et al., 2011; Bennett, 2011). Moreover, CIs are becoming commonplace in science education for quantifying student performance (see Sayre and Heckler, 2009; Nehm 2019).

**Assessing Contextual Reasoning about Natural Selection (ACORNS)**

The ACORNS is a constructed-response assessment that requires a student to generate expository responses to describe the evolutionary change in various settings presented. The student is graded on inclusion of normative key concepts (KCs) which include competition, differential survival, differential reproduction, heritability, limited resources, and non-adaptive reasoning, and penalized for evolutionary “misconceptions” or naive ideas (Nehm and Reilly, 2007). If a student includes only KCs in their responses, this student will receive a score of 1 for their ACORNS coherence model type. If a student uses a mixture of KCs and naïve terms, their score will be 2. If only naïve ideas are used, they will receive a score of 5. 1,131 observations (35.1%) are missing for these predictors (KCs and ACORNS coherence model type). Students’ responses were scored using EvoGrader’s machine learning algorithms (Moharreri et al., 2014). This computerized grading system has been shown to be equivalent to human scoring (Beggrow et al., 2014).

**Conceptual Inventory of Natural Selection** (**CINS)**

The CINS is a multiple-choice assessment that measures 10 evolutionary concepts. Each correct response yields a score of 1 and each incorrect response yields a score of 0 (Anderson et al., 2002). Each question has four answer choices with one correct answer and three incorrect answers. 1,107 CINS entries (34.3%) are missing.

These CI assessments were used to quantify a student’s knowledge of evolution prior to course commencement. The instructor (kept consistent for all six semesters) used these metrics as a course diagnostic tool.

**F: Overview of data pipeline steps**

Regardless of academic and research discipline, a computationally efficient and robust pipeline centers on data mining and knowledge discovery. When predicting students’ academic performances, educators need to consider various data amalgamation, data manipulation, and data mining steps in order to transform a corpus into a suitable format for analysis and rigorously evaluate the model’s predictive accuracy. The main stages of a robust and computationally feasible pipeline can be summarized in four steps.

**Step one: data manipulation**

Once educational stakeholders identify a target outcome to forecast, features must be identified and amalgamated from independent educational data sources into a single corpus. Past studies predicting student performance have focused on leveraging data from the prior academic achievements of students (e.g., high school grade point average [GPA], SAT score) (Rath et al., 2007; Dobson, 2008; Eddy et al., 2014). An increasing emphasis has been on incorporating institution-specific data with course-specific information from the current academic experiences of students (e.g., learning management systems (LMS), concept inventory (CI) assessments). These combined data sources have been shown to enhance the accuracy of prediction models (Minaei-Bidgoli et al., 2003; Lykourentzou et al., 2009; Wolff et al., 2014; Koprinska et al., 2015; Bertolini et al., 2021).

**Step two: data preprocessing**

Once merged, the single corpus must be cleaned and examined for any errors in the manipulation process or within the data itself. This is arguably the most important step in the pipeline (Chicco, 2017). This includes but is not limited to (1) detecting outliers, (2) transforming the data so the ranges of different features are comparable, (3) filling in missing data entries (imputation), (4) re-balancing the data if a class imbalance is present, and (5) converting categorical features into indicator variables (Li 2019). Depending on the data corpus, only a selection of these steps may be needed. Feature selection may also be performed as a preprocessing step of the pipeline.

**Step three: data modeling**

Model performance is inextricably linked to data preprocessing since the cleaned and manipulated data will serve as inputs to the DMMs. To tune the parameters for each DMM, several techniques (e.g., cross-validation or grid search) can be used for optimal hyperparameter selection on the training data. Often, researchers use several DMMs independently or combine them to see if maximum predictive performance can be achieved. These techniques are called ensemble methods; but they are not as interpretable as traditional predictive models (‘black-box methods’ - Rokach, 2010; Watson et al., 2019).

**Step four: model evaluation**

Following training, DMMs are evaluated on a testing corpus to assess predictive efficacy via performance metrics (e.g., AUC). Although the EDM literature has focused on identifying the features that impact DMM performance (see Literature Review), a comprehensive study assessing the features chosen by different feature selection algorithms in educational data pipelines has not to our knowledge been completed.

**G: Data pipeline for the collegiate biology classroom**

**Step one: data manipulation**

Data were aggregated from the institution’s data warehouse. We considered a set of diverse academic and non-academic factors in our pipeline (see Additional file 1: Material A for summary statistics and a complete list of all predictors used in this study).

**Step two: data preprocessing**

Various preprocessing steps were applied to the corpora:

*Convert Categorical Features to Indicator Variables*.  The 15 categorical variables in our corpora were converted to indicator variables using the ‘dummies’ package in R (R Core Team, 2017).

*Imputation.* The MICE (Multivariate Imputation via Chained Equations) package was used to impute missing data (Buuren & Groothuis-Oudshoorn, 2010). In particular, the predictive mean matching (PMM) regression technique was employed since it is useful for imputing covariates which are not necessarily normally distributed by matching observations with missing entries to observations without missing data entries (Allison 2015). Following the results of a simulation study conducted by Fox and Weisberg (2018), 50 iterations of this technique were used. MICE has become increasingly popular in the field of EDM (Hochweber et al., 2014; Belfi et al., 2016; Crespo-Turrado et al., 2016; Alexandro, 2018).

*Standardization and Rescaling of Features.* The assembled data features were on different scales from one another. For example, high school GPA ranged from 66 to 100 while mathematics placement examination scores ranged between one and nine. All features were transformed to a z-score by taking each feature, subtracting it by the feature mean, and dividing it by the feature standard deviation. This made the distribution of each feature have a zero mean and unit standard deviation.

*SMOTE*. Our classification problem involves a class imbalance in the dependent variable since there is a disproportionate number of students who passed (majority class) compared to those who failed (minority class). When imbalanced data are encountered, it is more difficult for prediction models to accurately predict students in the latter category (Weiss and Provost, 2003; Prati et al., 2004). In classification, balanced data records have been shown to increase the predictive accuracy of DMMs on independent corpora (Radwan and Cataltepe, 2017; Laureano et al., 2020). Due to its popularity in EDM (Kotsiantis, 2009; Márquez-Vera et al., 2011; Fernández-Delgado et al., 2014; Pujianto et al., 2020), the oversampling technique SMOTE was applied to rectify the class disparity in the training corpus (Chawla et al., 2002). The R default value of *k=5* nearest neighbors was used.

**Step three: data modeling**

During cross-validation and hyperparameter tuning, feature selection was applied.

*Ten-Fold Cross-Validation & Feature Selection*. Cross-validation was applied to tune the parameters for each DMM. The DMM with the tuning parameters obtained from the fold that had the highest AUC (see step four) was applied to the testing corpus to evaluate model performance in the pipeline (James et al., 2013). Ten folds were used in this study following the recommendation of Davison and Hinkley (1997).

During cross-validation, the importance of each feature was evaluated by applying a filter feature selection technique on each of the 10 training folds. Borda’s method was used and the $\log_{2} (m)$- cutoff was applied to select 57 = 5.83 ≈ 6 features to include in the final prediction model run on the testing corpus. While forecasts were generated using aggregated features at each time frame, six features were chosen using this cutoff for consistency across all training corpora.

**Step four: model evaluation**

Three metrics were used for pipeline evaluation: AUC metric, SC metric, and Jaccard index. The latter two methods were used to mathematically compare the top six features identified by the Borda tabulation.

*AUC Metric*. The primary metric to evaluate the predictive efficacy of the DMMs in this study was the AUC. The closer the AUC is to 1, the more accurate a DMM is at predicting student performance (Friedman et al., 2001).

*SC Metric*. To compare the stability of each feature selection technique with respect to each training corpus, we applied a measurement by Nogueira and Brown (2016) to subsets of the training data:

Equation 7: SC Metric

$$SC= \frac{2}{m(m-1)}\sum_{i=1}^{m-1} \sum_{j=i+1}^{m} Cor(z_{i},z_{j})$$

In equation 7, *m* is the number of subsets that the training corpus is divided into. $Cor(z_{i},z_{j})$ is the Pearson correlation coefficient measuring the association between the vectors $z_{i}$ and $z_{j}$. The *j*^th^ component of $z_{i}$ is one provided the *i*^th^ fold identifies feature $X_{j}$ as being relevant in modeling the target outcome. This metric takes on values between negative one and one, inclusively, where one denotes maximum stability and zero indicates that the feature selection technique is as stable as selecting features at random (Bommert et al., 2020).

*Jaccard Index*. The Jaccard Index was used to discern similarities between the top six features identified between each pair of preprocessing feature selection techniques. For feature selection techniques *A* and *B*, the Jaccard Index is defined as the number of features common to both techniques divided by the size of their union:

Equation 7: Jaccard Index

$$J\left( A,B \right)= \frac{|A\cap B|}{|A\cup B|}= \frac{|A\cap B|}{\left| A \right|+\left| B \right|-|A\cap B|}$$

The Jaccard Index takes on a value between zero and one (Jaccard 1901). Unlike other similarity metrics, such as the simple matching coefficient for binary outcomes which accounts for the presence and absence of a feature, the Jaccard Index only accounts for the presence of features (Rand, 1971). The latter method is more appropriate for our study since we are using this metric to compare whether the top six features selected by the preprocessing filter feature selection techniques, using the Borda tabulation, are similar.

**H: AUC regression model & ANOVA analysis for SC metric and Jaccard index**

**AUC Multiple Regression Model**

A multiple regression model (Table S16) was used to perform an analysis of the AUC by examining (1) testing semester, (2) size of the training corpus, (3) time frame, (4) DMM, and (5) preprocessing feature selection technique. All comparisons were conducted at a 1% level of significance (α = 0.01). Overall, the regression model explained 45.1% of the variability in the AUC results (multiple regression p-value < 0.0001).

Compared to when preprocessing feature selection techniques were omitted from the data science pipeline, FSA and CAE significantly increased the AUC, on average, by 0.058 (t-value = 6.435, p-value <.0001) and 0.040 (t-value = 4.391, p-value <.0001) points, respectively. RAE was the worst performing preprocessing feature selection technique and yielded AUC values that were, on average, 0.068 points lower compared to when feature selection was omitted (t-value = -7.501, p-value <.0001). There was no significant difference in the AUC values obtained when IG was applied (t-value = -1.012; p-value = 0.312).

The AUCs obtained for a fall testing semester were not significantly different from the AUC values obtained from spring testing semesters (t-value = -1.982, p-value = 0.048). Furthermore, the length of the training database did not significantly impact prediction success in our modified pipeline. Compared to models run using five semesters of training data, the use of two, three, and four semesters of prior course records reduced the AUC, on average, by 0.009, 0.024, and 0.009 points, respectively. The ensemble technique GLMNET significantly outperformed all other DMMs, indicated by the negative regression coefficients and p-values.

Table S16: Regression coefficient estimates and significance tests for the effect of (1) testing semester, (2) number of training semesters, (3) time frame, (4) data mining method, and (5) preprocessing feature selection technique

| **Independent Variable**  **(Base Comparison)** | **Estimated Coefficient** | **Standard Error** | **T**  **Value** | **P**  **Value** |
| --- | --- | --- | --- | --- |
| Intercept  Fall testing corpus (Spring testing corpus)  2 training corpora (5 training corpora)  3 training corpora (5 training corpora)  4 training corpora (5 training corpora)  Week 3 (Pre-course)  Week 6 (Pre-course)  Week 9 (Pre-course)  LR (GLMNET)  RF (GLMNET)  XGBoost (GLMNET)  CAE (no feature selection technique)  FSA (no feature selection technique)  IG (no feature selection technique)  RAE (no feature selection technique) | 0.762  -0.012  -0.009  -0.024  -0.009  0.065  0.084  0.133  -0.055  -0.073  -0.094  0.040  0.058  -0.010  -0.068 | 0.013  0.006  0.011  0.011  0.012  0.008  0.008  0.008  0.008  0.008  0.008  0.009  0.009  0.009  0.009 | 59.311  -1.982  -0.808  -2.262  -0.802  7.947  10.280  16.429  -6.759  -8.962  -11.523  4.391  6.435  -1.012  -7.501 | < .0001 ***  0.048  0.420  0.024  0.423  < .0001 ***  < .0001 ***  < .0001 ***  < .0001 ***  < .0001 ***  < .0001 ***  < .0001 ***  < .0001 ***  0.312  < .0001 *** |
| Significance codes: 0.001 ‘**’; < .0001 ‘***’  F-statistic: 47.83 on 14 and 785 degrees of freedom; p-value: < .0001  Residual Standard Error = 0.08  Adjusted R^2^ = 0.451  LR: Logistic Regression; GLMNET: Elastic Net Regression; RF: Random Forest; XGB: Extreme Gradient Boosting; CAE: Correlation Attribute Evaluation; FSA: Fisher’s Scoring Algorithm; IG: Information Gain Attribute Evaluation; RAE: Relief Attribute Evaluation | | | | |

**ANOVA Analyses for SC Metric and Jaccard Index**

Two ANOVA analyses were used to examine differences between the SC metric values and Jaccard indices obtained between each pair of preprocessing feature selection techniques. While a level of significance was set at 0.01, all post-hoc pairwise comparisons reported in Table S17 and Table S18 used Tukey’s honestly significant difference with adjusted p-values (Tukey 1949).

Across all prediction designs, the four preprocessing feature selection techniques yielded significantly different SC metric values (*F*_3,108_ = 1733, p-value < .0001). Except for the difference in the SC metric between CAE and FSA (t – ratio = -0.860, adjusted p-value = 0.8254), all pairwise differences were statistically significant.

Table S17: Tukey’s honestly significant difference multiple comparisons analysis for the SC metric between each pair of preprocessing feature selection techniques

| Contrast* | Estimate | T – Ratio | Adjusted P-Value |
| --- | --- | --- | --- |
| CAE - FSA | -0.0107 | -0.860 | 0.8254 |
| CAE - IG | 0.1029 | 8.255 | < .0001 *** |
| CAE - RAE | 0.7571 | 60.767 | < .0001 *** |
| FSA - IG | 0.1136 | 9.115 | < .0001 *** |
| FSA - RAE | 0.7679 | 61.627 | < .0001 *** |
| IG - RAE | 0.6543 | 52.512 | < .0001 *** |
| Significance codes: < .0001 ‘***’ | | | |

An analogous analysis was performed to compare differences between the Jaccard indices obtained for each pair of preprocessing filter feature selection techniques. Across all prediction designs, the four feature selection techniques yielded significantly different Jaccard indices (*F*_5,54_ = 40.30, p-value < .0001).

Table S18: Tukey’s honestly significant difference multiple comparisons analysis for the Jaccard index between each pair of preprocessing feature selection techniques

| Contrast* | Estimate | T – Ratio | Adjusted P-Value |
| --- | --- | --- | --- |
| CAE_FSA – CAE_IG | 0.5228 | 9.281 | < .0001 *** |
| CAE_FSA – CAE_RAE | 0.6446 | 11.443 | < .0001 *** |
| CAE_FSA – FSA_IG | 0.5437 | 9.652 | < .0001 *** |
| CAE_FSA – FSA_RAE | 0.6337 | 11.250 | < .0001 *** |
| CAE_FSA – IG_RAE | 0.6689 | 11.874 | < .0001 *** |
| CAE_IG – CAE_RAE | 0.1218 | 2.162 | 0.2721 |
| CAE_IG – FSA_IG | 0.0209 | 0.371 | 0.9990 |
| CAE_IG – FSA_RAE | 0.1109 | 1.969 | 0.3733 |
| CAE_IG – IG_RAE | 0.1461 | 2.593 | 0.1167 |
| CAE_RAE – FSA_IG | -0.1009 | -1.791 | 0.4798 |
| CAE_RAE – FSA_RAE | -0.0109 | -0.194 | 1.0000 |
| CAE_RAE – IG_RAE | 0.0242 | 0.430 | 0.9980 |
| FSA_IG – FSA_RAE | 0.0900 | 1.598 | 0.6036 |
| FSA_IG – IG_RAE | 0.1252 | 2.222 | 0.2450 |
| FSA_RAE – IG_RAE | 0.0352 | 0.624 | 0.9888 |
| Significance codes: < .0001 ‘***’ | | | |

*A_B denotes the Jaccard index for filter methods A and B, respectively.

**I: Additional Materials References**

Alexandro, D. (2018). *Aiming for Success: Evaluating Statistical and Machine Learning Methods to Predict High School Student Performance and Improve Early Warning Systems.* Ph.D. thesis, University of Connecticut, Storrs, CT.

Allison, P. (2015). Imputation by Predictive Mean Matching: Promise and Peril. *https://statisticalhorizons.com.*

Anderson, D.L., Fisher, K.M., & Norman, G.J. (2002). Development and evaluation of the conceptual inventory of natural selection. *Journal of research in science teaching, 39*(10), 952-978.

Beggrow, E.P., Ha, M., Nehm, R.H., Pearl, D., & Boone, W.J. (2014). Assessing scientific practices using machine-learning methods: How closely do they match clinical interview performance? *Journal of Science Education and Technology, 23*(1), 160-182.

Belfi, B., Haelermans, C., & De Fraine, B. (2016). The long-term differential achievement effects of school socioeconomic composition in primary education: A propensity score matching approach. *British Journal of Educational Psychology,* 86(4), 501-525.

Bennett, R.E. (2011). Formative assessment: a critical review. *Assessment in Education: Principles, Policy, & Practice, 18*(1), 5-25.

Bertolini, R., Finch, S.J., & Nehm, R.H. (2021). Testing the Impact of Novel Assessment Sources and Machine Learning Methods on Predictive Outcome Modeling in Undergraduate Biology. *Journal of Science Education and Technology, 30(2)*, 193-209.

Bolón-Canedo, V., Sánchez-Maroño, N., Alonso-Betanzos, A. (2013). A review of feature selection methods on synthetic data. *Knowledge and information systems, 34*(3), 483-519.

Bommert, A., Sun, X., Bischl, B., Rahnenführer, J., & Lang, M. (2020). Benchmark for filter methods for feature selection in high-dimensional classification data. *Computational Statistics & Data Analysis, 143*, 106839.

Buuren, S.V., & Groothuis-Oudshoorn, K. (2010). mice: Multivariate Imputation by Chained Equations in R. *Journal of Statistical Software, 45*(3), 1-68.

Chawla, N.V.M., Bowyer, K.W., Hall, L.O., & Kegelmeyer, W.P. (2002). SMOTE: synthetic minority over-sampling technique. *Journal of artificial intelligence research, 16*, 321-357.

Chicco, D. (2017). Ten quick tips for machine learning in computational biology. *BioData mining, 10*(1), 35.

Crespo-Turrado, C., Casteleiro-Roca, J.L., Sánchez-Lasheras, F., López-Vázquez, J.A., De Cos Juez, F.J., Calvo-Rolle, J.L., & Corchado, E. (2016). Student performance prediction applying missing data imputation in electrical engineering studies degree. In *International Conference on Hybrid Artificial Intelligence Systems*. Springer, Cham, 126-135.

Davison, A.C., & Hinkley, D.V. (1997). *Bootstrap Methods and their Application* (Volume 1). Cambridge University Press.

Dobson, J. L. (2008). The use of formative online quizzes to enhance class preparation and scores on summative exams. *Advances in Physiology Education, 32*(4), 297-302.

Duda, R.O., Hart, P.E., & Stork, D.G. (2003). *Pattern classification*. New York: Wiley-interscience.

Eddy, S.L., Brownell, S.E., & Wenderoth, M.P. (2014). Gender gaps in achievement and participation in multiple introductory biology classrooms. *CBE - Life Sciences Education, 13*(3), 478-492.

Fernández-Delgado, M., Mucientes, M., Vázquez-Barreiros, B., & Lama, M. (2014). Learning analytics for the prediction of the educational objectives achievement. In *2014 IEEE Frontiers in Education Conference (FIE) Proceedings*. IEEE, 1-4.

Fox, J., & Weisberg, S. (2018). *An R Companion to Applied Regression*. Sage Publications.

Friedman, J.H., Hastie, T., & Tibshirani, R. (2001). *The Elements of Statistical Learning (Volume 1, No. 10)*. New York: Springer.

Hall, M.A. (1999). *Correlation-based feature selection for machine learning*. Ph.D. thesis, University of Waikato, Hamilton, New Zealand.

Haudek, K.C., Kaplan, J.J., Knight, J., Long, T., Merrill, J., Munn, A., Nehm, R.H., Smith, M., & Urban-Lurain, M. (2011). Harnessing technology to improve formative assessment of student conceptions in STEM: forging a national network. *CBE – Life Sciences Education, 10*(2), 149-155.

Hochweber, J., Hosenfeld, I., & Klieme, E. (2014). Classroom composition, classroom management, and the relationship between student attributes and grades. *Journal of Educational Psychology, 106*(1), 289.

Holmes, G., Donkin, A., & Witten, I.H. (1994). Weka: A machine learning work bench. *Proceedings of the Second Australia and New Zealand Conference on Intelligent Information Systems*. Brisbane, Australia: Institute of Electrical and Electronics Engineers. IEEE, 357-361.

Jaccard, P. (1901). Distribution de la flore alpine dans le bassin des Dranses et dans quelques régions voisines. *Bulletin de Soci*été *Vaudoise des Sciences Naturelles 37*, 241-272.

James, G., Witten, D., Hastie, T., Tibshirani, R. (2013). *An Introduction to Statistical Learning* *(Vol. 112).* New York: Springer.

Laureano, L.B., Sison, A.M., & Medina, R.P. (2020). Affinity Propagation SMOTE approach for Imbalanced dataset used in Predicting Student at Risk of Low Performance. *International Journal, 9*(4), 5066-5070.

Li, C. (2019). Preprocessing Methods and Pipelines of Data Mining: An Overview. *arXiv preprint arXiv: 1906.08510.*

Lykourentzou, I., Giannoukos, I., Mpardis, G., Nikolopoulos, V., & Loumos, V. (2009). Early and dynamic student achievement prediction in e-learning courses using neural networks. *Journal of the American Society for Information Science and Technology, 60*(2), 372-380.

Kira, K., & Rendell, L.A. (1992). A practical approach to feature selection. In *Machine Learning Proceedings 1992*. Morgan Kaufmann, 249-256.

Koprinska, I., Stretton, J., & Yacef, K. (2015). Predicting student performance from multiple data sources. *International Conference on Artificial Intelligence in Education*. Springer, Cham, 678-681.

Kotsiantis, S. (2009). Educational data mining: a case study for predicting dropout-prone students. *International Journal of Knowledge Engineering and Soft Data Paradigms, 1*(2), 101-111.

Márquez-Vera, C., Romero, C., & Ventura, S. (2011). Predicting School Failure Using Data Mining. *4^th^ International Conference on Educational Data Mining.* Eindhoven, Netherlands, 271-275.

Mangal, A. (2018). *Applied Machine Learning to Predict Stress Hotspots in Materials*. Ph.D. thesis, Carnegie Mellon University, Pittsburgh, PA.

Minaei-Bidgoli, B., Kashy, D. A., Kortemeyer, G., & Punch, W. F. (2003). Predicting student performance: An application of data mining methods with an education web-based system. *33rd Annual* *Frontiers in Education, 2003. FIE 2003 Volume 1*. IEEE, T2A-13.

Moharreri, K., Ha, M., & Nehm, R.H. (2014). EvoGrader: an online formative assessment tool for automatically evaluating written evolutionary explanations. *Evolution: Education and Outreach, 7*(1), 1-14.

Nehm, R.H., Beggrow, E.P., Opfer, E.P., & Ha, M. (2012). Reasoning about natural selection: diagnosing contextual competency using the ACORNS instrument. *The American Biology Teacher, 74*(2), 92-98.

Nehm, R.H. (2019). Biology education research: building integrative frameworks for teaching and learning about living systems. *Disciplinary and Interdisciplinary Science Education Research, 1*(1), 1-18.

Nehm, R.H., & Reilly, L. (2007). Biology majors’ knowledge and misconceptions of natural selection. *BioScience, 57*(3), 263-272.

Nogueira, S., & Brown, G. (2016). Measuring the stability of feature selection. In *Joint European Conference on Machine Learning and Knowledge Discovery in Databases*. Springer, Cham, 442-457.

Prati, R.C., Batista, G.E., & Monard, M.C. (2004). Class imbalances versus class overlapping: an analysis of a learning system behavior. In *Mexican International Conference on Artificial Intelligence*. Springer, Berlin, Heidelberg, 312-321.

Pujianto, U., Prasetyo, W.A., & Taufani, A.R. (2020). Students Academic Performance Prediction with k-Nearest Neighbor and C4.5 on SMOTE-balanced data. In *2020 3rd International Seminar on Research of Information Technology and Intelligent Systems (ISRITI).* IEEE, 348-353.

Radwan, A., & Cataltepe, Z. (2017). Improving performance prediction on education data with noise and class imbalance. *Intelligent Automation & Soft Computing,* 1-8.

Rand, W.M. (1971). Objective criteria for the evaluation of clustering methods. *Journal of the American Statistical Association, 66*(336)*,* 846-850.

Rath, K., Peterfreund, A., Xenos, S., Bayliss, F., & Carnal, N. (2007). Supplemental instruction in introductory biology I: Enhancing the performance and retention of underrepresented minority students. *CBE- Life Science Education, 6*(3), 203-216.

R Core Team. (2017). R: A language and environment for statistical computing. Vienna, Austria: R Foundations for Statistical Computing.

Robnik-Šikonja, M., & Kononenko, I. (2001). Comprehensible interpretation of relief’s estimates. In *Machine Learning: Proceedings of the Eighteenth International Conference on Machine Learning (ICML2001).* Morgan Kaufmann*,* 443-440.

Rokach, L. (2010). Ensemble-based classifiers. *Artificial Intelligence Review, 33*(1-2), 1-39.

Sayre, E.C., & Heckler, A.F. (2009). Peaks and decays of student knowledge in an introductory E&M course. *Physical Review Special Topics – Physics Education Research, 5*(1), 013101.

Tang, J., Alelyani, S., & Liu, H. (2014). Feature selection for classification: A review. *Data classification: Algorithms and application, 37*, 1-29.

Tukey, J.W. (1949). Comparing individual means in the analysis of variance. *Biometrics, 5*(2), 99-114.

Urbanowicz, R.J., Meeker, M., La Cava, W., Olson, R.S., & Moore, J.H. (2018). Relief-Based Feature Selection: Introduction and Review. *Journal of Biomedical Informatics, 85*, 189-203.

Vege, S.H. (2012). *Ensemble of feature selection techniques for high dimensional data.* Ph.D. thesis, Western Kentucky University, Bowling Green, Kentucky.

Watson, D.S., Krutzinna, J., Bruce, I.N., Griffiths, C.E., McInnes, I.B., Barnes, M.R., & Floridi, L. (2019). Clinical applications of machine learning algorithms: beyond the black box. *Bmj, 364*.

Weiss, G.M. & Provost, F. (2003). Learning when training data are costly: The effect of class distribution on tree induction. *Journal of Artificial Intelligence Research, 19*, 315-354.

Wolff, A., Zdrahal, Z., Herrmannova, D., & Knoth, P. (2014). Predicting student performance from combined data sources. In *Educational Data Mining*. Springer, Cham, 175-202.
